# A comprehensive benchmarking study of protocols and sequencing platforms for 16S rRNA community profiling

**DOI:** 10.1186/s12864-015-2194-9

**Published:** 2016-01-14

**Authors:** Rosalinda D’Amore, Umer Zeeshan Ijaz, Melanie Schirmer, John G. Kenny, Richard Gregory, Alistair C. Darby, Migun Shakya, Mircea Podar, Christopher Quince, Neil Hall

**Affiliations:** Institute of Integrative Biology, University of Liverpool, Liverpool, L69 7ZB UK; School of Engineering, University of Glasgow, Glasgow, G12 8LT UK; Department of Biological Sciences, Dartmouth College, Hanover, NH03755 USA; Biosciences Division, Oak Ridge National Laboratory, Oak Ridge, 37831 TN USA; Warwick Medical School, University of Warwick, Warwick, CV4 7AL UK

## Abstract

**Background:**

In the last 5 years, the rapid pace of innovations and improvements in sequencing technologies has completely changed the landscape of metagenomic and metagenetic experiments. Therefore, it is critical to benchmark the various methodologies for interrogating the composition of microbial communities, so that we can assess their strengths and limitations. The most common phylogenetic marker for microbial community diversity studies is the 16S ribosomal RNA gene and in the last 10 years the field has moved from sequencing a small number of amplicons and samples to more complex studies where thousands of samples and multiple different gene regions are interrogated.

**Results:**

We assembled 2 synthetic communities with an even (EM) and uneven (UM) distribution of archaeal and bacterial strains and species, as metagenomic control material, to assess performance of different experimental strategies. The 2 synthetic communities were used in this study, to highlight the limitations and the advantages of the leading sequencing platforms: MiSeq (Illumina), The Pacific Biosciences RSII, 454 GS-FLX/+ (Roche), and IonTorrent (Life Technologies). We describe an extensive survey based on synthetic communities using 3 experimental designs (fusion primers, universal tailed tag, ligated adaptors) across the 9 hypervariable 16S rDNA regions. We demonstrate that library preparation methodology can affect data interpretation due to different error and chimera rates generated during the procedure. The observed community composition was always biased, to a degree that depended on the platform, sequenced region and primer choice. However, crucially, our analysis suggests that 16S rRNA sequencing is still quantitative, in that relative changes in abundance of taxa between samples can be recovered, despite these biases.

**Conclusion:**

We have assessed a range of experimental conditions across several next generation sequencing platforms using the most up-to-date configurations. We propose that the choice of sequencing platform and experimental design needs to be taken into consideration in the early stage of a project by running a small trial consisting of several hypervariable regions to quantify the discriminatory power of each region. We also suggest that the use of a synthetic community as a positive control would be beneficial to identify the potential biases and procedural drawbacks that may lead to data misinterpretation. The results of this study will serve as a guideline for making decisions on which experimental condition and sequencing platform to consider to achieve the best microbial profiling.

**Electronic supplementary material:**

The online version of this article (doi:10.1186/s12864-015-2194-9) contains supplementary material, which is available to authorized users.

## Background

Over the last 5 years improvements in sequencing technologies have seen the launching of the Single Molecule, Real-Time (SMRT$^{\circledR }$) DNA Sequencing System from Pacific Bioscience; and benchtop sequencers from 454, Life Technologies (now Thermo), and Illumina. These technologies have completely changed the way the scientific community have designed and conceived experiments in microbial ecology to describe community complexity. With the rapid reduction in the cost of sequencing and improvements in read length and throughput, the field has been completely revolutionized and experimental designs, that before could not even have been attempted due to the high cost involved, have been made achievable. Two approaches have been widely used in practice to describe microbial community structure, 16S rRNA gene profiling and shotgun metagenomic sequencing.

Ribosomal RNA genes are highly conserved and evolutionarily stable but differ in their hypervariable region, these features have made them the ideal tool for phylogenetic studies. Several papers have described the natural variation using *in silico* analyses [1] and genome sequencing have boosted our knowledge of the biological world improving ribosomal gene databases which support phylogenetic studies. These improvements have made the 16S rRNA gene the ideal marker for the characterization of microbial community diversity [2]. The 16S rRNA gene compromises 9 hypervariable regions, which differ in length, position and taxonomic discrimination [3]. Universal primers have been designed and evaluated to amplify the hypervariable regions [4] and primer bias toward particular taxonomic groups has been reported [5, 6]. The variable regions have different discriminatory power depending on the groups of microbes and amongst the short target regions (<300 bp), the hypervariable region 4 (V4) was generally the most informative [7].

In the last 5 years the most commonly used next generation sequencing platform for amplicon sequencing has been the 454 series of platforms (Roche) [8, 9] and many groups have developed bioinformatics pipelines and denoising algorithms [10–12] that make the 454 a robust approach to investigate microbial diversity. However, Roche have recently announced the withdrawal of the 454 sequencing platforms by 2016 and this has highlighted the need for an alternative for taxonomic studies. The various sequencing platforms available have different strengths and weaknesses in read-length, accuracy, time-to-result, and throughput. Longer reads are easier to assign to a taxonomic group because they contain more information but some recent studies have suggested that it is possible to achieve comparable results using shorter target regions [4, 13] and overlapping reads have been employed [14] to increase the taxonomic discrimination on Illumina platforms.

Also, depending on the community being studied and the nature of the hypotheses posed, different target regions and multiplexing strategies can be employed. Therefore, it is likely that different groups will opt for different study designs. Hence, it is crucial to develop technical solutions and bioinformatics pipelines that can be applied across multiple study designs.

Microbial profiling studies the abundance and the type of organisms within an environmental community. As with most molecular methods, a profiling experiment requires a series of distinct steps, which can potentially introduce errors and may even lead to biases and incorrect findings [15–17].

Even though natural microbial communities are composed of a mix of microbes with unknown structure, synthetic microbial communities can be assembled to generate distinct systems with reduced complexity [18–20]. Previous studies have reported the use of a synthetic community as a control material to: investigate biases; interrogate different software packages; and find the impact of sample preparation, 16S rRNA primer choice, amplicon preparation, direct sampling and library preparation [15, 18–21]. In this study we used two synthetic communities assembled *in vitro* by mixing genomic DNA from 49 bacterial and 10 archaeal species at equal abundance (even community EM) or uneven abundance (uneven community UM as shown in Table 1).

**Table 1:** Composition of synthetic communities with biological classification of the organisms as well as the proportions used for UM community in this study

ID	Genome name	Genome size (bp)	Domain	Phylum	Class	Proportion UM community (%)
ACI_CAP	*Acidobacterium capsulatum ATCC 51196*	4127356	Bacteria	Acidobacteria	Acidobacteriae	8.1
AKK_MUC	*Akkermansia muciniphila ATCC BAA-835*	2664102	Bacteria	Verrucomicrobia	Verrucomicrobiae	0.9
ANE_THE	*Anaerocellum thermophilum Z-1320*, DSM 6725	2919718	Bacteria	Firmicutes	Clostridia	1.2
BAC_THE	*Bacteroides thetaiotamicron VPI-5482*	6293399	Bacteria	Bacteroidetes	Bacteroidia	0.2
BAC_VUL	*Bacteroides vulgatus ATCC 8482*	5163189	Bacteria	Bacteroidetes	Bacteroidia	0.9
BOR_BRO	*Bordetella bronchiseptica RB50*	5339179	Bacteria	Proteobacteria	Betaproteobacteria	9.2
BUR_XEN	*Burkholderia xenovorans LB400*	973113	Bacteria	Proteobacteria	Betaproteobacteria	2.6
CAL_SAC	*Caldicellulosiruptor saccharolyticus DSM 8903*	2970275	Bacteria	Firmicutes	Clostridia	2
CHL_TEP	*Chlorobaculum tepidum TLS*	2154946	Bacteria	Chlorobi	Chlorobia	0.5
CHL_LIM	*Chlorobium limicola DSM 245*	2763181	Bacteria	Chlorobi	Chlorobia	0.4
CHL_PHA226	*Chlorobium phaeobacteroides DSM 266*	3133902	Bacteria	Chlorobi	Chlorobia	1.9
CHL_PHA265	*Chlorobium phaeovibrioides DSM 265*	1966858	Bacteria	Chlorobi	Chlorobia	0.3
CHL_AUR	*Chloroflexus aurantiacus J-10-fl*	5258541	Bacteria	Chloroflexi	Chloroflexi	0.9
CLO_THE	*Clostridium thermocellum ATCC 27405*	3843301	Bacteria	Firmicutes	Clostridia	0.6
DEI_RAD	*Deinococcus radiodurans R1*	3284156	Bacteria	Thermi	Deinococci	1.7
DES_DES	*Desulfovibriodesulfuricans ATCC 27774*	2873437	Bacteria	Proteobacteria	Deltaproteobacteria	1.4
DES_PIG	*Desulfovibrio piger ATCC 29098*	2826240	Bacteria	Proteobacteria	Deltaproteobacteria	3.1
DIC_TUR	*Dictyoglomus turgidum DSM 6724*	1855560	Bacteria	Dictyoglomi	Dictyoglomia	3.5
ENT_FAE	*Enterococcus faecalis V583*	3359974	Bacteria	Firmicutes	Bacilli	4.3
FUS_NUC	*Fusobacterium nucleatum ATCC 25586*	2174500	Bacteria	Fusobacteria	Fusobacteria	0.3
GEM_AUR	*Gemmatimonas aurantiaca T-27T*	4636964	Bacteria	Gemmatimonadetes	Gemmatimonadetes	0.7
HER_AUR	*Herpetosiphon aurantiacus ATCC 23779*	6785430	Bacteria	Chloroflexi	Chloroflexi	1.8
HYD_Y04AAS1	*Hydrogenobaculum sp. Y04AAS1*	1559514	Bacteria	Aquificae	Aquificae	1.1
LEP_CHO	*Leptothrix cholodnii SP-6*	4909403	Bacteria	Proteobacteria	Betaproteobacteria	1.8
NIT_EUR	*Nitrosomonas europaea ATCC 19718*	2812094	Bacteria	Proteobacteria	Betaproteobacteria	4.3
NOS_PCC7120	*Nostoc sp. PCC 7120*	7211789	Bacteria	Cyanobacteria	unclassified	2.7
PEL_PHA	*Pelodictyon phaeoclathratiforme BU-1*	3018238	Bacteria	Chlorobi	Chlorobia	0.1
PER_MAR	*Persephonella marina EX-H1*	2467104	Bacteria	Aquificae	Aquificae	5.5
POR_GIN	*Porphyromonas gingivalis ATCC 33277*	2354886	Bacteria	Bacteroidetes	Bacteroidia	0.2
RHO_BAL	*Rhodopirellula baltica SH 1*	7145576	Bacteria	Planctomycetes	Planctomycetacia	1
RHO_RUB	*Rhodospirillum rubrum ATCC 11170*	4406557	Bacteria	Proteobacteria	Alphaproteobacteria	1.2
RUE_POM	*Ruegeria pomeroyi DSS-3*	4601053	Bacteria	Proteobacteria	Alphaproteobacteria	0.6
SAL_ARE	*Salinispora arenicola CNS-205*	5786361	Bacteria	Actinobacteria	Actinobacteria	0.5
SAL_TRO	*Salinispora tropica CNB-440*	5183331	Bacteria	Actinobacteria	Actinobacteria	1.6
SHE_BAL_OS185	*Shewanella baltica OS185*	5312910	Bacteria	Proteobacteria	Gammaproteobacteria	3.1
SHE_BAL_OS223	*Shewanella baltica OS223*	5358884	Bacteria	Proteobacteria	Gammaproteobacteria	1.4
SUL_EE.36	*Sulfitobacter sp. EE-36*	3547243	Bacteria	Proteobacteria	Alphaproteobacteria	2
SUL_NAS.14.1	*Sulfitobacter sp. NAS-14.1*	4002069	Bacteria	Proteobacteria	Alphaproteobacteria	4.3
SUL_YO3AOP1	*Sulfurihydrogenibium sp. YO3AOP1*	1838442	Bacteria	Aquificae	Aquificae	1.6
SUL_YEL	*Sulfurihydrogenibium yellowstonense SS-5*	1534471	Bacteria	Aquificae	Aquificae	2.6
THE_PSE	*Thermoanaerobacter pseudethanolicus ATCC 33223*	2362816	Bacteria	Firmicutes	Clostridia	0.8
THE_NEA	*Thermotoga neapolitana DSM 4359*	1884562	Bacteria	Thermotogae	Thermotogae	0.7
THE_PET	*Thermotoga petrophila RKU-1*	1824357	Bacteria	Thermotogae	Thermotogae	1
THE_RQ2	*Thermotoga sp. RQ2*	877693	Bacteria	Thermotogae	Thermotogae	3.4
THE_THE	*Thermus thermophilus HB8*	2116056	Bacteria	Thermi	Thermi	0.5
TRE_DEN	*Treponema denticola ATCC 35405*	2843201	Bacteria	Spirochaetes	Spirochaetes	0.2
TRE_VIN	*Treponema vincentii I*	2512734	Bacteria	Spirochaetes	Spirochaetes	0.2
ZYM_MOB	*Zymomonas mobilis mobilis ZM4*	2223497	Bacteria	Proteobacteria	Alphaproteobacteria	0.8
ARC_FUL	*Archaeoglobus fulgidus DSM 4304*	2178400	Archaea	Euryarchaeota	Archaeoglobi	0.3
IGN_HOS	*Ignicoccus hospitalis KIN4/I*	1297538	Archaea	Crenarchaeota	Thermoprotei	1.2
MET_JAN	*Methanocaldococcus jannaschii DSM 2661*	1664970	Archaea	Euryarchaeota	Methanococci	0.9
MET_MAR_C5	*Methanococcus maripaludis C5*	1780761	Archaea	Euryarchaeota	Methanococci	0.4
MET_MAR_S2	*Methanococcus maripaludis S2*	1661137	Archaea	Euryarchaeota	Methanococci	0.5
NAN_EQU	*Nanoarchaeum equitans Kin4-M*	490885	Archaea	Nanoarchaeota	Nanoarchaea	1
PYR_AER	*Pyrobaculum aerophilum IM2*	2222430	Archaea	Crenarchaeota	Thermoprotei	0.5
PYR_CAL	*Pyrobaculum calidifontis JCM 11548*	2009313	Archaea	Crenarchaeota	Thermoprotei	2.6
PYR_HOR	*Pyrococcus horikoshii OT3*	1738505	Archaea	Euryarchaeota	Thermococci	1.9
SUL_TOK	*Sulfolobus tokodaii 7(S311)*	2694756	Archaea	Crenarchaeota	Thermoprotei	0.7

Different amplicon library preparation methodologies have been proposed to unlock the power of next-gen technologies for targeted sequencing [22–26]. The fusion primer design (FP) uses PCR to attach a barcode [22] but is expensive due to the requirement of purchasing long primers that are platform specific for every combination of primer and barcode to pair each sample in a study (e.g., 96 samples × 4 variable regions would require a total of 384 primer pairs).

The adaptor ligase (AD) approach ligates a barcoded adapter to the end of an amplicon. The AD approach uses a platform specific library preparation kit instead of specially designed fusion primers, and is functional for experiments where amplicons already exists or at least where template-specific primers already exist [24]. The universal tailed-tag amplicon design (t-tag) uses a slightly more elaborate two-step library amplification process but it is more economical, avoiding the cost of a large number of barcoded primers [27]. This approach reduces the number of primers needed by attaching, in a first round of PCR, the linker/universal primer to each end and requires only as many primer pairs as the number of variable regions in the experiment. A second round of PCR is carried out, with universal primers hybridizing to the linker while the indexing sequence can be added to one or both ends to barcode the samples. As the second set of primers can be reused in other experiments (with different targets) this method can be much cheaper than other approaches. Illumina and most recently PacBio have developed a protocol based on the 2 step PCR design and a similar approach has been supported by Fluidigm Corporation [26].

**Fig. 1:**
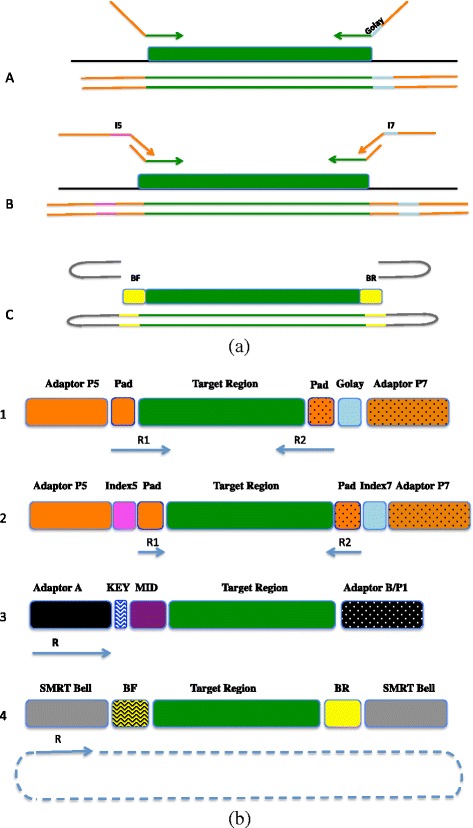
Experimental design. (**a**) Design of single and dual-index sequencing strategy and schematic describing the 3 amplicon designs: Fusion Primer Design (A) is a one step PCR which uses a single 12-nt error-correcting Golay index sequence (blue) allowing a high multiplexing capability. Tag tailed design (B) is a 2-step PCR which uses a universal primer for the first step and a dual index barcoded primer set in the second step. Standard Illumina Nextera 8-nt index sequences were used (pink Index 5; blue Index 7). The Pac Bio Ligate Adapters design (C): Two harpin adapters (grey) were ligated to a barcoded template (BF forward barcode; BR reverse barcode) to allow multiplexing. (**b**) Platform Specific Amplicon Libraries: Illumina paired-end sequencing (1,2) generates 2 sequencing reads (R1 and R2) per each cluster and can have single (Standard/Golay) or dual indexes (I5, I7). Ion Torrent and 454 (3) have a single read for each bead with a single index (MID). Pacific Bioscience generate a single circular read for each molecule (SMRT bell) and can have one (BF or BR) or two indexes. The starting point and direction of sequencing reads are indicated by a solid blue line and arrows, respectively. In the case of Fusion Primer Design custom sequencing primer were used

**Fig. 2:**
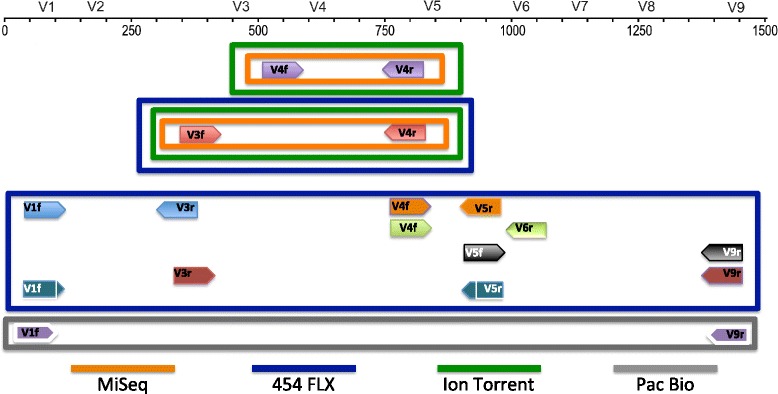
Schematic representation of the combination of primers covering the 16S rRNA hypervariable regions and the sequencing platform used in this study

We compared the fusion primer (FG) and tailed tag (t-tag) methodologies (Fig. 1) for building amplicon libraries using the Illumina MiSeq platform. A single or a dual indexing strategy was also evaluated. Moreover, we generated amplicon libraries across the 9 hypervariable regions of the 16S rRNA gene (Fig. 2) for 2 synthetic communities and compared the data generated with a shotgun metagenomic library. Amplicon libraries were sequenced on 454 GS FLX/FLX+, Illumina MiSeq (MS), Life Technologies Ion Torrent (IT) and Pacific Bioscience RS II (PB) platforms, to determine the impact of different experimental conditions on the community structure.

Our results demonstrate that almost all aspects of experimental design will bias the results to some extent. We demonstrate that not only specific target region affected the community profiling but also PCR cycle numbers, sequencing platform, and library preparation method will give different results in terms of error rates and biases. Despite this, by comparing data from UM and EM communities we show that the data on all sequencing platforms is quantitative even if it does not accurately describe the community composition. We also highlight the relative merits of the different experimental designs for describing species within a community and OTU estimation.

## Methods

### Even and uneven microbial mock communities

High-molecular weight DNA was extracted using a mechanical and organic cell lysis method as described in [28], dissolved in TE buffer (pH 8) and measured using a Qubit$^{\circledR }$ dsDNA BR assay. After quantification and calculation of the concentration of genomic copies for each DNA preparation, two mixtures of genomic DNAs were assembled, henceforth referred to as even (EM) and uneven (UM) synthetic communities. We considered organisms that were either sequenced previously or had a high quality draft genome in NCBI repository and covered a wide variety of strains that can be found in marine and terrestrial environments. Unlike natural environmental communities where composition and abundance is unknown, the two synthetic communities were assembled to have known amount of purified gDNA from *Archaea* and *Bacteria* domains. Both communities comprised ten members of *Euryarcheota*, *Crenarcheota* and *Nanoarcheota* for *Archaea* and 49 bacterial strains from 16 phyla. The same organisms were used previously [28] although they have been assembled in a different manner in this paper. Our emphasis is on exploring the impact of the distributions of abundance distributions on recovery diversities. Therefore, we assembled an EM community to aim for an equal number of molecules per strain and an UM community where the strains within the same phylum were distributed according to a log-normal distribution with proportions shown in Table 1. In the UM, proportion between bacterial and archaeal strains was 9:1.

### Amplicon library designs

The conditions studied varied across a gradient of template concentrations at a high cycle number and a low cycle number. We performed 3 independent amplifications using HiFi Hot Start polymerase (Kapa) or Q5 polymerase (New England BioLabs) to test for polymerase derived bias (Table 2). Kapa and NEB have developed PCR amplification kits specifically for NGS workflows and these are widely used as they have high yields with little amplification bias. The 2 kits were selected for their ability to amplify difficult templates (e.g AT- and GC- rich) and increase yield, speed and sensitivity.

**Table 2:** Experimental conditions assessed in this study on IT, MS, 454 FLX + and PB sequencing platforms

Synthetic community	Experimental design	Nm cycles	Template concentration (ng)	Sequencing platform
EM/UM	Fusion primer	25	1,5,10	MS
EM	Fusion primer	25	2,5	IT, 454
EM	Universal tailed tag	5+15	1,10	MS
EM	Universal tailed tag	8+15	1,10	MS
EM/UM	Universal tailed tag	10+15	2,5	MS
EM/UM	Adapter ligation	25	500–750	PB

#### Universal tailed tag design (single or dual index barcoding strategy)

1−10 ng of EM or UM community DNA was used in the first amplification step using the following conditions: 0.1 *μ*M forward tailed target specific primer; 0.1 *μ*M reverse tailed target specific primer; and 1x HiFi or Q5 polymerase ready mix. The PCR for each variable region was carried out in triplicate in a 25 *μ*l reaction in a thermal cycler (Applied Biosystem GeneAmp PCR system 9700) with the following parameters: initial denaturation at 94 °C for 5 mins, followed by 5, 8, and 10 cycles of 98 °C for 20 s, 60 °C for 15 s, and 72 °C for 40 s with a final extension at 72 °C for 1 min. The amplicon libraries were cleaned to remove excess nucleotides, salts and enzymes using 20 *μ*l of the Agencourt AMPure XP system (Beckman Coulter Genomics) and eluted in 10 *μ*l of TE buffer. The 10 *μ*l of the first step reaction was submitted to a second amplification step using the following conditions: 0.1 *μ*M forward barcoded primer for the dual index strategy or a forward not barcoded primer for the single index strategy; 0.1 *μ*M primer barcoded reverse primer; 1x HiFi (Kapa) or Q5 (NEB) polymerase ready mix. The PCR for each variable region was carried out in triplicate in a 25 *μ*l reaction in the above-mentioned thermal cycler with the following parameters: initial denaturation at 94 °C for 5 min, followed by 15 cycles of 98 °C for 20 s, 60 °C for 15 s, and 72 °C for 40 s with a final extension at 72 °C for 1 min.

#### Fusion primer design

1−10 ng of the EM or UM community was subjected to an amplification step using 0.3 *μ*M primer forward fusion specific; 0.3 *μ*M primer reverse fusion specific; 1x HiFi or Q5 polymerase ready mix. The PCR for each variable region was carried out in triplicate in a 25 *μ*l reaction in the thermal cycler with the following parameters: initial denaturation at 94 °C for 5 min, followed by 25 cycles of 98 °C for 20 s, 60 °C for 15 s, and 72 °C for 40 s with a final extension at 72 °C for 1 min.

#### Adapter ligation design

2 ng of the EM community DNA was submitted to the following amplification reaction: 0.3 *μ*M forward variable region specific primer; 0.3 *μ*M reverse variable region specific primer; and 1x HiFi polymerase ready mix. The PCR for each variable region was carried out in triplicate in a 25 *μ*l reaction in the thermal cycler with the following parameters: initial denaturation at 94 °C for 5 min, followed by 25 cycles of 98 °C for 20 s, 60 °C for 15 s, and 72 °C for 40 s with a final extension at 72 °C for 1 min. The amplicon libraries were cleaned to remove excess nucleotides, salts and enzymes using 20 *μ*l of the Agencourt AMPure XP system. 1 *μ*l of each PCR product was run on a Bioanalyzer HS DNA Chip to ensure the final product was the correct size and quantified using a Qubit$^{\circledR }$ dsDNA HS assay. A pool of the three independent PCR products (500−750 ng) were subjected to end-repair using the Pacific Biosciences DNA template prep kit 2.0. (250 bp - 3 k) following manufacturer recommendations. Briefly, the end repair reaction was incubated at 25 °C for 15 min. Following the purification with 0.6x of AMPure PB beads and elution in 30 *μ*l of elution buffer, the end repaired mixtures were blunt ligated with the adapters supplied in the template prep kit. The ligation was incubated at 25 °C for 15 min and then stored at 4 °C overnight. Exonuclease digestion to remove the failed ligation products was performed by incubating at 37 °C for 1 h, followed by purification with 1x AMPure beads.

### Amplicon quantitation and pooling

1 *μ*l of each amplicon library was run on a Bioanalyzer HS DNA Chip to assess the amplicon size and quantified using a Qubit$^{\circledR }$ dsDNA HS assay. Each amplicon library was size selected according to the expected amplicon size (±50 bp) using a pre-cast 1.5 % agarose with ethidium bromide gel cassette on the Pippin Prep System (Sage Science). The library concentration for Illumina and 454 amplicon libraries was assessed using a SYBR green qPCR assay with primers specific to each platform (Kapa).

### Multiplexing strategy

Three multiplexing approaches were used to tag each PCR product: single index, dual index, and barcoded adapter. The single index and dual index strategies were used to build libraries for Illumina’s MS technology. In the single index strategy approach, the primers contain the Illumina adapter sequence, a unique 12nt error-correcting Golay index sequence (only for the reverse primer; see Table 3) comprising a 10nt pad to prevent hairpin formation, and a 2nt linker that is not complementary to the 16S rRNA gene and a gene specific sequence. Sequencing proceeded by using the combined pad-linker-primer as sequencing primers at the $3^{\prime }$ and $5^{\prime }$ ends (read 1 and read 2 sequence primers are shown in Table 4) as described in Caporaso et al. [29]. In the dual index strategy, the forward and reverse locus specific primers are modified to include a 35nt Illumina adapter sequence at the $5^{\prime }$ ends that act as primer binding sites in the second step PCR (Table 4). In the second step PCR, universal tag primer set (Table 4) which contains a 39nt sequence (corresponding to the Illumina adapter needed for cluster generation) comprising a unique 8nt index sequence (Table 5), and a 21nt complementary to the sequence introduced in the first step PCR, to tag the the library produced in the first step. The single index strategy was used to build libraries suitable for IT and 454 sequencing. In the single index strategy approach the primers contain the IT/454 adapter sequence, a 4nt key sequence, a unique 10nt index sequence (only for the forward primer; see Table 6) and a gene specific sequence. The ligate adapter strategy was used to build libraries to submit to PB sequencing. Forward and reverse locus specific primers were used to amplify the variable region but the primers were modified to include a 5nt pad sequence and a unique 16nt index sequence (Table 7) at the $5^{\prime }$ ends. After amplification, PB specific adapters were ligated to both ends.

**Table 3:** The error-correcting multiplex identifier sequences used with MS technology. A 12 bp reverse index used for unidirectional tagging in the fusion primer approach (F)

Name	Type	Design	Sequence
806rcbc0	Golay	F	TCCCTTGTCTCC
806rcbc1	Golay	F	ACGAGACTGATT
806rcbc2	Golay	F	GCTGTACGGATT
806rcbc3	Golay	F	ATCACCAGGTGT
806rcbc4	Golay	F	TGGTCAACGATA
806rcbc5	Golay	F	ATCGCACAGTAA
806rcbc6	Golay	F	GTCGTGTAGCCT
806rcbc7	Golay	F	AGCGGAGGTTAG
806rcbc8	Golay	F	ATCCTTTGGTTC
806rcbc9	Golay	F	TACAGCGCATAC
806rcbc10	Golay	F	ACCGGTATGTAC

**Table 4:** PCR primers used in this study

Primer name	Platform	Library design	Variable region	Sequence
454_27YMF	454	F	V1-V3	CCATCTCATCCCTGCGTGTCTCCGACTCAG**xxxxxxxxxx** ***AGAGTTTGATYMTGGCTCAG***
454_515R				CCTATCCCCTGTGTGCCTTGGCAGTCTCAG**TTACCGCGGCKGCTGNCAC**
454_F341	454	F	V3-V4	CCATCTCATCCCTGCGTGTCTCCGACTCAG**xxxxxxxxxxCCTAYGGGRBGCASCAG**
454_816R1				CCTATCCCCTGTGTGCCTTGGCAGTCTCAG**GGACTACHVGGGTWTCTAAT**
454_F515	454	F	V4-V5	CCATCTCATCCCTGCGTGTCTCCGACTCAG**xxxxxxxxxxGTGNCAGCMGCCGCGGTAA**
454_926R				CCTATCCCCTGTGTGCCTTGGCAGTCTCAG**CCGYCAATTYMTTTRAGTTT**
454_F515	454	F	V4-V6	CCATCTCATCCCTGCGTGTCTCCGACTCAG**xxxxxxxxxxGTGNCAGCMGCCGCGGTAA**
454_1061R				CCTATCCCCTGTGTGCCTTGGCAGTCTCAG**CRRCACGAGCTGACGAC**
454_F515	454	F	V4	CCATCTCATCCCTGCGTGTCTCCGACTCAG**xxxxxxxxxxGTGNCAGCMGCCGCGGTAA**
454_816R1				CCTATCCCCTGTGTGCCTTGGCAGTCTCAG**GGACTACHVGGGTWTCTAAT**
454_F515A	454	F	V4A	CCATCTCATCCCTGCGTGTCTCCGACTCAG**xxxxxxxxxxGTGBCAGCMGCCGCGGTAA**
454_805RA				CCTATCCCCTGTGTGCCTTGGCAGTCTCAG**GACTACHVGGGTATCTAATCC**
454_F787	454	F	V5-V9	CCATCTCATCCCTGCGTGTCTCCGACTCAG**xxxxxxxxxxATTAGATACCCNGGTAG**
454_1492R				CCTATCCCCTGTGTGCCTTGGCAGTCTCAG**TACGGYTACCTTGTTAYGACTT**
1Round515For	MS	DI	V4	CTACACTCTTTCCCTACACGACGCTCTTCCGATCTNNNNN**GTGCCAGCMGCCGCGGTAA**
1Round806Rev				GTGACTGGAGTTCAGACGTGTGCTCTTCCGATCT**GGACTACHVGGGTWTCTAAT**
1RounN515F	MS	DI	V4	CTACACTCTTTCCCTACACGACGCTCTTCCGATCT**GTGCCAGCMGCCGCGGTAA**
1Round806R				GTGACTGGAGTTCAGACGTGTGCTCTTCCGATCT**GGACTACHVGGGTWTCTAAT**
1Round341For	MS	DI	V4	CTACACTCTTTCCCTACACGACGCTCTTCCGATCT**CCTAYGGGRBGCASCAG**
1Round805RARev				GTGACTGGAGTTCAGACGTGTGCTCTTCCGATCT**GACTACHVGGGTATCTAATCC**
1Round515AFor	MS	DI	V4	CTACACTCTTTCCCTACACGACGCTCTTCCGATCT**GTGBCAGCMGCCGCGGTAA**
1Round805RARev				GTGACTGGAGTTCAGACGTGTGCTCTTCCGATCT**GACTACHVGGGTATCTAATCC**
DI_N5XXFor	MS	DI	V4	AATGATACGGCGACCACCGAGATCTACACxxxxxxxx**ACACTCTTTCCCTACACGACG**
DI_N7xxRev				CAAGCAGAAGACGGCATACGAGATxxxxxxxx**GTGACTGGAGTTCAGACGTGTGCTCTTCCGATCT**
FG515for	MS	F	V4	AATGATACGGCGACCACCGAGATCTACACTATGGTAATTGT**GTGCCAGCMGCCGCGGTAA**
FG8xxrev				CAAGCAGAAGACGGCATACGAGATxxxxxxxxxx**AGTCAGTCAGCCGGACTACHVGGGTWTAAT**
Read 1 Seq Primer	MS	F	V4	TATGGTAATTGTGTGCCAGCMGCCGCGGTAA
Read 2 Seq Primer	MS	F	V4	AGTCAGTCAGCCGGACTACHVGGGTWTCTAAT
Index Seq Primer	MS	F	V4	ATTAGAWACCCBDGTAGTCCGGCTGACTGACT
454_F341	IT	F	V3-V4	CCATCTCATCCCTGCGTGTCTCCGACTCAGCxxxxxxxxxx**CCTAYGGGRBGCASCAG**
TtP1_Kn805rev				CCTCTCTATGGGCAGTCGGTGATGGACTACHVGGGTWTCTAAT
454_F515A	IT	F	V4	CCATCTCATCCCTGCGTGTCTCCGACTCAGxxxxxxxxxx**GTGBCAGCMGCCGCGGTAA**
TtP1_Kn805rev				CCTCTCTATGGGCAGTCGGTGATG**GACTACHVGGGTATCTAATCC**
454_F515	IT	F	V4	CCATCTCATCCCTGCGTGTCTCCGACTCAGACATACGCGTGTGNCAGCMGCCGCGGTAA
TtP1_Kn806rev				CCTCTCTATGGGCAGTCGGTGATG**GGACTACHVGGGTWTCTAAT**
PBv1F	PB	LA	V1-V9	ggtagxxxxxxxxxxxxxxxx**AGAGTTTGATYMTGGCTCAG**
PBv9R				ccatcxxxxxxxxxxxxxxxx**TACGGYTACCTTGTTAYGACTT**

The variable region primer sequence is displayed in bold. The position of the multiplex identifier (MID) is shown as [x] and the respective sequences are shown in Tables 3, 5, 6, and 7. Degenerated bases in the sequence are represented as follows: M: C or A; B: not A; Y: C or T; R: A or G; W: A or T; H: not G; K: G or T; V: not T

**Table 5:** Unique barcode adaptors specifically designed and validated for optimal performance with Illumina technology

Name	Type	Design	Sequence
501	Illumina	DI	TAGATCGC
502	Illumina	DI	CTCTCTAT
503	Illumina	DI	TATCCTCT
504	Illumina	DI	AGAGTAGA
505	Illumina	DI	GTAAGGAG
506	Illumina	DI	ACTGCATA
507	Illumina	DI	AAGGAGTA
508	Illumina	DI	CTAAGCCT
701	Illumina	DI	TCGCCTTA
702	Illumina	DI	CTAGTACG
703	Illumina	DI	TTCTGCCT
704	Illumina	DI	GCTCAGGA
705	Illumina	DI	AGGAGTCC
706	Illumina	DI	CATGCCTA
709	Illumina	DI	AGCGTAGC
710	Illumina	DI	CAGCCTCG
711	Illumina	DI	CAGCCTCG

An 8 bp reverse Index (I7) and forward index (I5) used in the universalTailed Tag design (DI) to barcode the reads in both directions

**Table 6:** Unique barcode adaptors specifically designed and validated for optimal performance with IT, 454 FLX and FLX+ sequencing technologies

Name	Type	Design	Sequence
TC20	454	F	ACGACTACAG
TC21	454	F	CGTAGACTAG
TC22	454	F	TACGAGTATG
TC23	454	F	TACTCTCGTG
TC24	454	F	TAGAGACGAG
TC25	454	F	TCGTCGCTCG
TC26	454	F	ACATACGCGT

A forward 10 bp MID was used in the fusion approach (F) to tag the reads in forward direction

**Table 7:** Unique barcode adaptors specifically designed and validated for optimal performance with PB sequencing technology. A forward and a reverse 16 bp MID was used in the Ligation approach (LA) to tag the reads in both directions

Name	Type	Design	Sequence
F12	PB	LA	CGCATCGACTACGCTA
R13	PB	LA	TGAGTAGCATGACACG
R14	PB	LA	GACATGCAGTCTCACA
R15	PB	LA	CAGTAGCGCACTGAGC
R16	PB	LA	CTGCGTGCGCGATAGT
R17	PB	LA	CGCGTGCAGAGTGTCA
R18	PB	LA	ATATCAGTCACGTCTG

### Metagenomic DNA Library

The metagenomic library was constructed using the EM community gDNA and the Illumina Nextera XT Kit. A standard tagmentation reaction was set up using 1ng as input according to the Nextera protocol. After neutralization, barcoded primers were added to the reaction and submitted to 12 cycles of amplification. After this, a PCR cleanup was performed following the Nextera protocol using a 0.6:1 ratio of AMPure XP$\circledR $ (Beckman Coulter) to PCR reaction. Reactions were eluted in 30 *μ*L of TE buffer.

### MS sequencing

Due to the low library diversity, a PhiX control spike-in of 10−15 % was used for libraries run with RTA v1.17.28, which is bundled with MCS v2.2. When the older version of the software was used, a PhiX control spike-in was added at 50 %. Each amplicon library was mixed with Illumina-generated PhiX control libraries and denatured with NaOH and subsequently the ssDNA library fragments were diluted to a final concentration of 8 pM. 600 *μ*l of ssDNA library was loaded into a MiSeq Reagent Cartridge and a 500–cycle PE kit v2 was used. Paired-end sequencing run was performed according to the manufacturer’s instruction (Illumina, San Diego, CA, USA). For the runs where fusion primer design and Golay barcodes were employed, we used custom read 1, read 2 and index read (see Table 4) according to [29]. Raw fastq files generated by the real time analysis software on the MS were used in the subsequent analyses.

### IT sequencing

IT sequencing was performed on the Ion Torrent Personal Genome Machine (PGM; Life Technologies, USA) according to the manufacturer’s protocols. 13 pM of size-selected libraries were amplified by PCR that was carried out using the Ion OneTouch^TM^ 200 Template Kit v2 DL (Life Technologies) according to the manufacturer’s instructions. Sequencing of the amplicon libraries was carried out on a 316 or 318 chip using the Ion Torrent PGM system and the Ion Sequencing 300 kit (Life Technologies) according to the supplier’s instructions. After sequencing, the individual sequence reads were filtered by the IT software to remove low quality and polyclonal sequences using default setting. All IT quality-approved, trimmed and filtered data were exported as Standard Flowgram Format (sff) files and used in subsequent analyses.

### sequencing

The libraries were clonally amplified via emulsion PCR adding 0.5 molecule/bead per cup of emulsion, following manufacturer’s recommendations employing the GS FLX Titanium LV emPCR Kit (454 Life Sciences, Branford, CT). Following amplification, emPCR reactions were collected, and emulsions broken according to the manufacturer’s protocols. Beads containing sufficient copies of clonally amplified library fragments were selected via the enrichment procedure and counted with a Z2 Coulter Counter (Beckman Coulter, Fullerton, CA) prior to sequencing. Following emulsion PCR enrichment, beads produced using the titanium library were deposited into 4-region gasket format wells of a Titanium Series PicoTiterPlate device and 454 sequencing was performed using the GS FLX Titanium Sequencing Kit XLR70 on the GS or using GS FLX Titanium Sequencing Kit XL+ on the GS FLX+ sequencer according to the manufacturer’s recommendations (454 Life Sciences, Branford, CT). Image analysis, signal processing and base calling were performed using the supplied software system. Sff files output from base calling were employed in downstream analyses using onboard software v2.6 for GS FLX and v2.8 for GS FLX+.

### PB SMRT sequencing

The Pacific Biosciences calculator was used to determine the amount of primers and polymerase needed for the binding reactions, based on an insert size of 1480 bp. The primers and Pacific Biosciences proprietary p4 SA DNA polymerase v2 was bound to the library and the MagBead Kit was used to bind the library complex with MagBeads before sequencing to reduce adapter dimers. The MagBeads SMRT bell-polymerase complexes were loaded into a 96 well plate. The plate, along with a DNA sequencing kit 2.0, was loaded onto the instrument. Each SMRT cell was loaded with a single binding complex and 180 min movies were collected. The run was demultiplexed using the read of insert pipeline on the onboard software provided in the smrt portal (software v2.1) applying as filtering 1, 3, 5, 8 passes and minimum predicted accuracy of 90.

### Bioinformatics

The results in this paper were generated using AMPLImock (Additional file 1: Figure S1; https://bitbucket.org/umerijaz/amplimock/src), a pipeline we developed for quantifying error rates and biases when a mock community with known reference sequences has been sequenced. The pipeline requires the original (F.fasta) and reverse-complimented (R.fasta) reference sequences for each 16S rRNA operon present and a mapping file (IDs.txt) which maps multiple 16S rRNA operons onto the known species.

#### Creation of the reference 16S rRNA database

For the compilation of the 16S rRNA reference database an *E. coli* 16S rRNA sequence (GI:349736152) was aligned against the full genome reference database using blastn [30]. If less than four blast hits were returned for any organism, the NCBI database was directly searched for additional sequences. For each individual organism duplicates and sequences that completely overlapped were removed. All reference sequences were subsequently verified by aligning them against the full genome reference database. Sequences that failed to align were removed, resulting in a total of 116 rRNA sequences. For the identification of single nucleotide polymorphisms (SNPs) in the 16S rRNA reference sequences, a large metagenomic sequencing data set (76 million reads, HiSeq, Nextera, 2 × 100 bp) in combination with a full length 16S sequencing data set (MiSeq, Nextera, 2 × 250 bp) was utilised. Two sequencing data sets were used in order to avoid the incorporation of false positive SNPs. Quality-trimmed reads with a minimum read length of 60 bp were aligned against the preliminary 16S rRNA reference database with BWA [31]. The alignment was subsequently converted to pileup format using SAMtools [32] and SNPs were identified with VarScan [33] (parameters for full length 16S data set: –min-var-freq 0.3–min-coverage 1000–strand-filter0 –variants; parameters for metagenomic data set: –min-var-freq 0.3 –min-coverage 80 –strand-filter 0 –variants). Only SNPs that were identified for both sequencing datasets were incorporated into the database through the addition of a new sequence containing the SNP. The process was repeated with the updated 16S reference database until no more SNPs were recognised for the metagenomic read data set. In total 33 SNPs were identified resulting in 128 16S rRNA reference sequences (note that if multiple SNPs were identified for the same reference sequence, one new sequence was added containing these SNPs).

#### Read trimming and filtering

The first step in the pipeline is filtering and quality trimming. For the IT, 454 and MS platforms, reads were filtered and quality trimmed with sickle (v1.200) which applies a sliding window approach and trims regions when the average base quality drops below 20 [34]. We also applied a 10 bp length threshold discarding reads that fall below this length after trimming. For the PacBio reads circular consensus sequencing (CCS) error correction was applied with different minimum thresholds (none i.e. raw reads of insert ROI, three - CCS3, five - CCS5 and eight - CCS8).

#### Generating non-chimeric overlapping reads

For the MS platform where paired-end reads were generated, we used PANDAseq (v2.4) with a minimum overlap of 50 bp to assemble them. PANDAseq was used as it has been previously shown to perform better than other software for paired-end assembly [35], reducing substitution rates for the MS platform by 77−98 *%* with an average of 93.2 *%*. Additionally, for the datasets where the raw data still had primers intact, we supplied primers to PANDAseq after assembling the reads to remove them from the resulting sequences. The overlapped reads were then dereplicated using uclust (v6.0.307), *de novo* clustered, annotated with cluster sizes, sorted while maintaining a record of redundancy and finally filtered for chimeras using uchime. Both initial overlapping reads, and final non-chimeric reads were then matched against the reference database (with minimum percentage identity of 95 %) to generate frequencies for each species in the reference database, error rates for matched reads and percentage of reads matching. For the IT, 454 and PB platforms where paired-end reads are not available, the overlapping step was skipped, and the sequences are searched against the reference database after initial QC checks using sickle.

#### OTU generation

We use the UPARSE (v7.0.1001) pipeline (http://www.drive5.com/usearch/manual/uparse_pipeline.html) for OTU generation. For the MS datasets, input to UPARSE are the overlapped reads generated from PANDAseq with AMPLImock, whereas for the rest of the platforms we use the single-end fasta files. The general approach is as follows: we pooled the reads from different samples together and added barcodes to keep an account of the samples these reads originate from. We then dereplicated the reads, sorted them by decreasing abundance and discarded singletons. In the next step, the reads are clustered based on 97 % similarity discarding reads that are shorter than 32bp. Even though the cluster_otu command in usearch removes reads that match chimeric models built from more abundant reads, a few chimeras may be missed, especially if they have parents that are absent from the reads or are present with very low abundance. Therefore, in the next step, we use a reference-based chimera filtering step using the GOLD database (http://drive5.com/uchime/uchime_download.html) that is derived from the ChimeraSlayer reference database in the Microbiome Utilities provided by the Broad Institute (http://microbiomeutil.sourceforge.net/). Finally, the original barcoded reads were matched against the clean OTUs with 97 % similarity to generate OTU tables for different samples. All these steps are mentioned in UPARSE.pdf located at https://bitbucket.org/umerijaz/amplimock/src. We have adopted two different approaches for generating OTU tables for each sample. In the first approach, we perform multiple sequence alignment of the database sequences using muscle (v3.8.31) and then excise the amplicon region based on the forward and reverse primers used for each sample with clustalw (v2.1). We then follow the general approach as mentioned before to generate a two column abundance table giving counts of OTUs that are present in the sample as well as the database. In the second approach, we generate the OTUs without collating the database sequences.

#### Generating pipeline statistics

AMPLImock generates quantitative results for each sample that can further be analysed in a statistical software package such as R. The total numbers of forward, reverse, and overlapped reads; and average PHRED quality scores of forward and reverse reads are outputted. These reads are then matched against the the reference 16S rRNA database extracted from the known genomes. From this the mean identity of forward (after trimming), reverse (after trimming), and overlapped reads, compared to their closest database match are calculated. The number of chimeric reads found in the above chimera checking steps and non-chimeric reads after chimera removal are also given. Finally, diversity indexes such as Shannon and Simpson index based on the proportion of different matched species in the database are calculated. Additionally, to determine error transition probabilities, we used the alignments against the best matching reference sequences generated by usearch to count all nucleotide transitions between the query and reference sequences.

## Results

### Error rates and percentage of reads matching across platforms

**Fig. 3:**
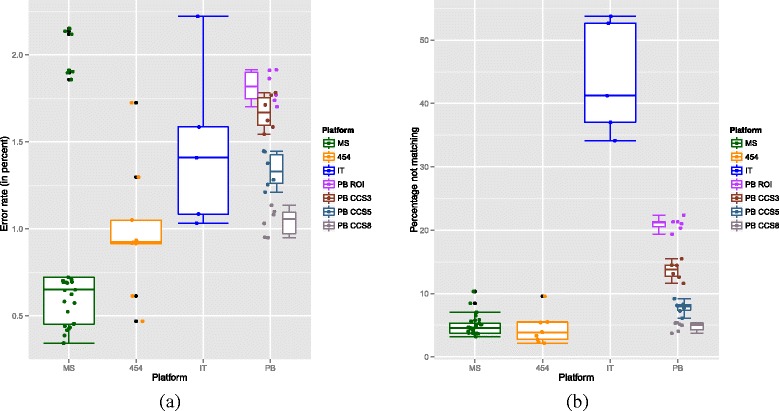
**a** Error rates across four different platforms. Platform had a significant impact on error rate (Kruskal-Wallis comparing MS, 454, IT and PB ROI non-parametric ANOVA *p*=0.015) as did number of CCS cycles for PB (*p*=0.016). **b** Percentage of reads not matching across the four different platforms. Platform had a significant impact on percentage matching (Kruskal-Wallis non-parametric ANOVA *p*=0.0001) as did number of CCS cycles for PB (*p*=0.016)

Error rates were calculated by matching reads against the reference 16S rRNA sequences using usearch as described above. Only reads with greater than 95 % nucleotide similarity to the best matching reference were used in the error calculations [36]. Results were calculated for all the EM community datasets with greater than 10,000 reads for the 454, IT and MS platforms but for PB, we used all data sets regardless of read number. This comprised 9 454 data sets, 5 FLX and 4 FLX+, 5 IT, 29 MS (see Table 8), and 3 PB replicates (each at four levels of CCS). To facilitate a fair comparison across all platforms, only forward reads were used for the MS platform, as although it allows paired-end sequencing, generating a forward and reverse read, this is not possible on the other platforms. In addition, the proportion of reads matching to the references was calculated. Overall, tests of significance were performed using a Kruskal-Wallis non-parametric ANOVA and individual t-tests performed to compare pairs of treatments. Error rates differed across platforms (see Fig. 3a) with an overall significance of *p*=0.015. The PB consensus sequences were generated using a multi-pass sequencing. The Read-of-Insert (ROI), i.e. unfiltered consensus reads with no minimum coverage, had the highest error rate of 1.90 % but this was not significantly higher than the IT - 1.47 % (*p*=0.11). Both the PB ROI and IT error rates were significantly higher than the MS (0.92 %) and 454 (1.06 %) but PB could be reduced to an equivalent error rate of 1.11 % when a minimum threshold of 8 passes (CCS8) was used.

**Table 8:** Experimental design parameters for MS EM datasets

Region	Amplicon design method	Primer (f)	Primer (r)	Input (ng)	PCR cycle no.	Taq	No.
V4	DI	515	805RA	2	12 + 18	HF	1
V4	DI	515	806rcb	2	12 + 18	HF	1
V4	DI	515	806rcb	2	10 + 15	HF	3
V4	DI	F515A	806rcb	2	8 + 15	HF	1
V4	DI	F515A	806rcb	2	8 + 15	Q5	3
V4	DI	F515A	806rcb	2	10 + 15	HF	3
V4	FG	515	806rcb	1	25	HF	3
V4	FG	515	806rcb	5	15	Q5	2
V4	FG	515	806rcb	5	25	Q5	2
V4	FG	515	806rcb	5	25	HF	1
V4	FG	515	806rcb	10	15	HF	1
V4	FG	515	806rcb	10	25	HF	2
V3-V4	DI	341f	806rcb	2	10 + 15	HF	3
V3-V4	DI	341f	805RA	2	10 + 15	HF	3

In calculating these error rates we only used reads with at most 5 % error; reads that are noisier than this will fail to match and hence not contribute to the error calculation. It is important therefore to also examine the percentage of reads that fail to match. This varied dramatically with platform (*p*=0.0001) as can be seen in Fig. 3b. The non-matching rate was similar for MS and 454 (means 4.83 % and 4.33 % respectively – *p*=0.56) but much higher for PB ROI (21.58497 %) and higher still for IT (mean 43.77 %). Once again, increased numbers of circularisation cycles reduced the PB percentage not matching to the range observed for MS and 454 albeit at the cost of reduced read number (see Additional file 2: Figure S2).

### Nature of errors across the different platforms

The nature of the observed errors differed across the different platforms too. In Additional file 3: Figure S3, we give heatmaps for each platform reflecting the proportion of the different possible transitions from the true ‘target’ based on the observed erroneous ‘query’ base. We include both base substitutions and insertions, a gap in the target sequence, or deletions, a gap in the query. The most common error for MS are substitutions and these are quite base-dependent with the transitions (A to G, G to A and T to C) being the most frequently observed, whereas for the other platforms insertions and deletions were more prevalent.

### Illumina: Impact of library preparation method and overlapping reads on error rate

**Fig. 4:**
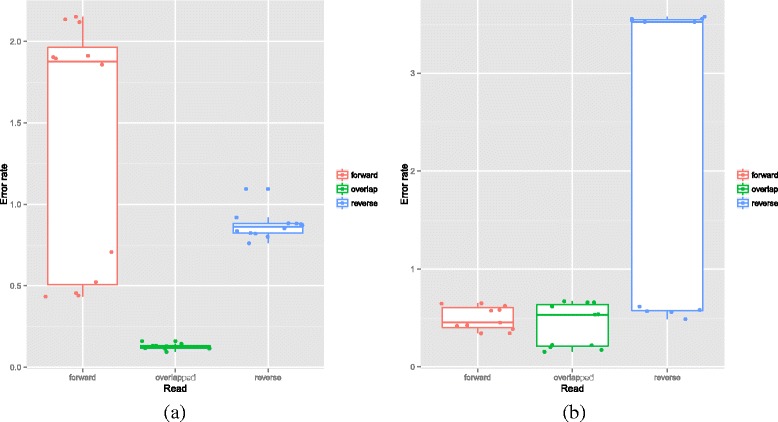
**a** Impact of overlapping reads on MS error rates for the DI library preparation method. Overlapping reads significantly reduced error rates for the DI library preparation method (t-test comparing forward [mean 1.38 %] and overlapped error rates [0.13 %] *p*=0.00016). **b** Impact of overlapping reads on MS error rates for the FG library preparation method. Overlapping reads did not significantly reduce error rate for the FG library preparation method (t-test comparing forward [mean 0.50 %] and overlapped error rates [0.42 %] *p*=0.36). It is also worth mentioning here that not all the reads overlapped, for example, for the MS platform, and with the given settings in PANDAseq (as discussed in the main text), the statistics for the percentage of reads that were assembled successfully are: 80.93 *%* (1^*st*^ quantile); 89.02 *%* (median); 81.07 *%* (mean); and 95.67 *%* (3^*rd*^ quantile)

We explored two alternative methods for building the Illumina MS libraries, the first involved a 1 step PCR with Golay barcodes on the reverse read (Fusion Primer Golay Design - FG) and the second a 2 step PCR with standard Illumina barcodes on both reads (Universal Tailed Tag Dual Index - DI) - see Fig. 1a, b. In Fig. 4a and b we show the impact of overlapping the forward and reverse reads on the MS V4 error rates. It is apparent that the DI method results in a significant reduction in the error rate compared to the FG. There is also a large variation in error rate for the forward DI reads and the reverse FG. This variation is highly run-dependent (see Additional file 4: Figure S9) but does not seem to depend on the cluster density of the particular run.

### Impact of PCR conditions on error rate and chimera frequency

**Fig. 5:**
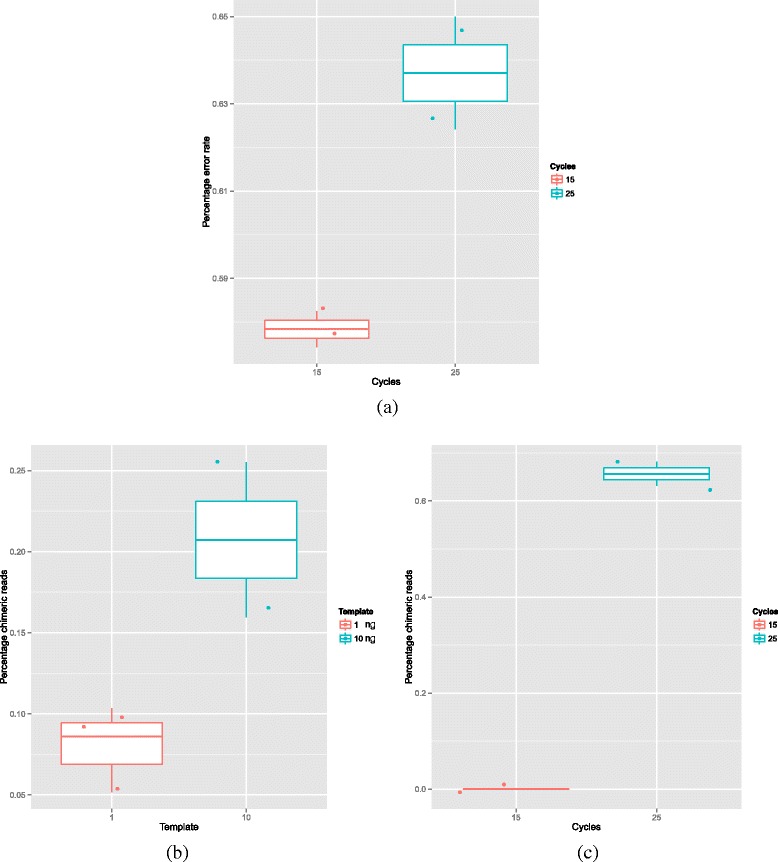
**a** Impact of no. of PCR cycles on the forward MS error rate. Increasing number of cycles did increase forward error rate with marginal significance for the FG library preparation method with Q5 Taq (t-test 15 cycles [mean 0.58 %] vs 25 cycles [mean 0.64 %] *p*=0.11). **b** Impact of PCR starting amount on percentage of chimeric reads. Decreased starting amount reduced percentage of chimeras for the FG library preparation method with HiFi Taq but not significantly (t-test comparing 1 ng [mean 0.08 %] and 10 ng [mean 0.2 %] *p*=0.20). **c** Impact of no. of PCR cycles on the percentage of chimeric reads. Increasing cycle number increased the percentage of chimeric reads for the FG library preparation method with Q5 Taq (t-test 15 cycles [mean 0.00 %] vs 25 cycles [mean 0.66 %] *p*=0.0245)

We also evaluated the impact of PCR conditions on the error rate. We explored the impact of starting template concentration, Taq polymerase enzyme and number of PCR cycles. Each sample was generated from the EM community using 3 independent PCR reactions targeting the V4 region of the 16S rRNA gene on MS using 2×250 bp paired-end reads. The only consistent effect observed on error rate was a marginally significant increase associated with more PCR cycles (*p*=0.11 - see Fig. 5a).

There is another form of artefact associated with PCR, in addition to simple base errors, and that is PCR chimeras. PCR chimeras are sequences comprised of two or more true sequences. They form due to incompletely extended sequences acting as primers to template in the following rounds of PCR. Our analysis revealed that the amount of starting material, and the number of cycles play an important role in controlling PCR errors. Using 1 ng of template reduced the proportion of chimeric reads compared to 10 ng but only with marginal significance (*p*=0.22) (see Fig. 5b). In contrast the cycle number did have a significant impact (*p*=0.0245 see Fig. 5c).

### Ability of different platforms and regions to reconstruct the EM community

**Fig. 6:**
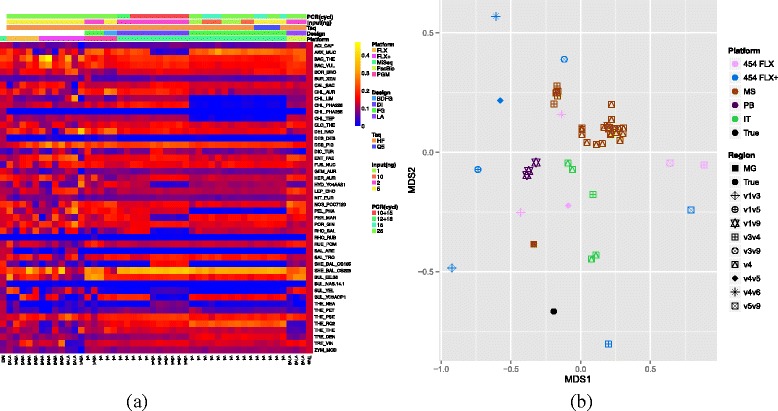
**a** Heatmap for EM communities (showing the bacterial species) reconstructed from different platforms using a range of experimental designs for amplicons. The design parameters are shown on top (**b**) NMDS plot based on Bray-Curtis distance comparing the samples showns in (**a**)

The observed species frequencies for the EM community varied with the choice of platform and sequenced region (see Fig. 6a). The individual species frequencies were highly unbalanced despite this community being designed to have equal molecule numbers for each genome. The causes of these biases could include primer mismatch, 16S rRNA copy number and amplification bias associated with the target length. The observed community associated with each primer platform combination is summarised in a two-dimensional non-metric multidimensional scaling visualisation in Fig. 6b. We also include a metagenome sample as a benchmark since this should be relatively unbiased given the fewer PCR amplification steps in the library construction. From this we see that the 454 FLX+ V4-V5 sample is closest to the metagenome. Other FLX+, FLX and PB samples also perform well probably reflecting their greater read length. The best MS region appears to be the V4. To quantify the effectiveness of the different platforms and regions with a single number we calculated Shannon’s entropy across species, i.e. $H(\bar {x}) = \sum _{s=1}^{S} - f_{s}\text {ln} (x_{s})$ where *x*_*s*_ is the observed relative frequency of species *s*. This value will be maximised for an even distribution when *x*_*s*_=1/*S* and therefore the higher the entropy, the closer the sample appears to the true underlying even community. The results are shown in Additional file 5: Figure S4a. This appears to confirm the NMDS plot in that one of the best performing combinations is 454 FLX+ V4-V5 although now the best of the MS V4 samples appears comparable, although there is a great deal of variation.

### Barcode switching

There is another form of error and that is barcode switching between labelled samples. To test for switching we prepared libraries from individual genomes rather than the mock communities. Then the frequency of observed reads deriving from species other than the focal one indicates the probability of barcode switching. We did this for both Illumina library preparation strategies. In both cases the overall switching rates were low with a mean switching probability of 0.17 % for DI and 0.21 % for FG with no significant difference between them (*p*=0.642 Additional file 6: Figure S5). The two library preparation strategies did differ in that for the DI most switching was to the other single species libraries on the run whereas for the FG a wider range of species from throughout the mock community was observed (see Additional file 7: Figure S6). In neither case was the mock community included on that run hence this may suggest that the FG library preparation method is more susceptible to carry-over from previous runs.

### Reconstruction of EM and UM communities

**Fig. 7:**
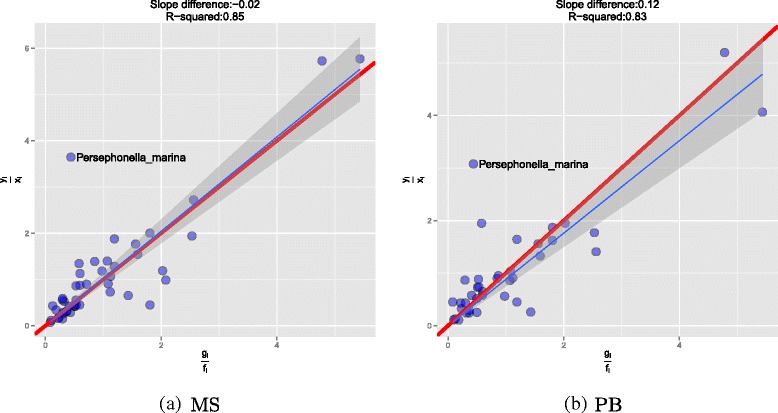
Quantitative results for two EM-UM pairs (among a total of 22) for MS and PB are shown. The fitted line through the points is represented by a blue line with R-squared shown on top. The red line is the ground-truth with the slope difference from the blue line also shown on top

We have demonstrated above that all platforms and regions suffer from substantial bias. The observed relative frequencies do not reflect the true species frequencies in the community. However, it is still possible that when we compare two samples, the observed differences between samples reflect the true differences. Hence, method is biased but still quantitative. To test this, we considered pairs of samples consisting of one from the EM and one from the UM community. Pairs were taken from the same run and this was performed for the MS (13 pairs) and PB (9 pairs) platforms. For each species *s* the true ratio of frequencies in the pair is known this is simply the ratio of the volumes used to generate the mixtures. We denote this *f*_*s*_/*g*_*s*_ where *f* and *g* are the relative frequencies in the UM and EM communities respectively. This is given on the x-axes of the plots in Fig. 7 and is compared to the ratio of the observed frequencies in the two samples for the same species, *y*_*s*_/*x*_*s*_. In Fig. 7 we give two examples one for the MS and one for the PB. In both cases there is a highly significant correlation between the two ratios and a slope that is nearly one for a regression forced through the origin. This implies that 16S rRNA sequencing is strongly quantitative despite being biased. In general the MS (mean R-squared of 0.8107) runs seem to be more quantitative than PB (mean R-squared of 0.716) with *p* = 0.044 using a t-test. We can then ask which species are responsible for this difference and which are more accurately quantified on one platform relative to another, by comparing the absolute errors, i.e. how different was the observed ratio from the true ratio? The results are given in Table 9. In all cases where there was a difference in accuracy, then the MS was the better platform. The exception was *Shewanella baltica OS223* which has a closely related strain in the data set *Shewanella baltica OS185*. This suggests that the one advantage of PB may be better strain resolution when the entire 16S rRNA is sequenced.

**Table 9:** Species with significantly different quantification accuracies between MS and PB

Species	Mean error MS	Mean error PB	*p*-value
*Caldicellulosiruptor*	0.132	0.643	0.000056966
*saccharolyticus*			
*Nitrosomonas*	0.510	1.117	0.000592824
*europaea*			
*Pelodictyon*	0.012	0.459	0.000072079
*phaeoclathratiforme*			
*Salinispora*	0.330	0.662	0.000132560
*tropica*			
*Shewanella*	0.560	0.207	0.000018219
*baltica OS223*			

### Taxonomic classification of reads to RDP database

Up until now we have calculated all statistics through comparison of the reads to the known 16S rRNA sequences of the species that we sequenced. This was useful to explore bias in community proportions but in reality we will not know *a priori* which organisms are present. Therefore, to provide a more realistic perspective of how diversity estimates and community predictions will be distorted we also performed *de novo* taxonomic classification against the RDP database using their standalone classifier [37] with the default –minWords option of 5. For the MS platform, assembled paired-end reads (through PANDAseq) were considered, whereas for other platforms, single-end reads were used. The results for observed genera number are shown in Additional file 8: Figure S7 where we have separated genera, by whether they were in the reference database and should be observed in the reads which we denote ‘good’, and also ‘noisy’ which are those genera which should not have been present. The short read platforms all outperformed PB in terms of their ability to recover the known genera, probably due to a combination of higher read number and less biased primers. Given this though they performed similarly although marginally more genera were recovered by MS than 454 (mean 39.305 and 37.454 respectively with *p*-value = 0.06029). However, 454 had fewer noisy genera than MS (mean 29.272 vs. 55.583 with *p*-value = 0.0009208) and fewer still than IT (mean 65.857 with *p*-value = 4.186e-05). The PB long reads also generated significantly fewer noisy OTUs than all the next generation technologies with a mean of 12.666.

### OTU construction

We next constructed OTUs from the reads from each platform using UPARSE with default parameters. We did not employ any of the denoising methods available for 454 (e.g. [10]) in order to provide an equivalent cross-platform comparison. We did discard singletons as recommended in UPARSE but not for the PB sequencing where the sample sizes were so small that the singletons contained a large proportion of the true diversity. In Additional file 9: Figure S8 we give the observed total OTU frequencies and of those ‘good’ OTUs that were also in the database and the ‘noisy’ OTUs that were not meant to be there. The full-length PB platform does succeed in identifying more good OTUs than the short read platforms, although not significantly more, but at the cost of far more ‘noisy’ artefact sequences. Of the short read platforms 454 and MS appear almost equivalent. Significantly more good OTUs were obtained for MS than IT (means 44.15 and 35.50, respectively, with *p*-value = 0.0001501) and significantly fewer noisy OTUs (means 18.83 and 6.2000 with *p*-value = 0.00538).

## Discussion

For any experiment it is vital to be able to understand the accuracy of the measurements and the potential sources of error. Here we have undertaken an exhaustive study that uses a multitude of primer combinations, library preparation protocols, and sequencing platforms. We quantified intrinsic errors and analyzed the relative accuracy of these approaches for absolute and relative estimation of species abundance and OTU estimation.

Our finding demonstrates the MS platform, using overlap read error correction, has the most accurate sequence reads. Despite this, the extra read length of the 454 platform and PB does allow good estimation of the composition of our mock community when comparing the data to the known reference dataset used in the study. However, given the much higher throughput and economics of the Illumina technology it would seem the pragmatic choice of platform for most studies.

As expected the number of PCR cycles during amplicon generation has a direct impact on the accuracy of the resulting data, we also demonstrate that the initial concentration of template will also affect the proportion of chimeras formed if the PCR cycle number is kept constant. This is most likely because when more template is present at the start of the reaction the amplicon abundance will increase more rapidly which enables more miss-priming during the PCR [21]. This demonstrates that normalizing the input DNA quantity across all samples is vital to ensure that all data generated is comparable.

It has been demonstrated by others that amplicon libraries can be prone to barcode switching [38, 39] whereby the barcodes from one amplicon can be assigned to another in the same flowcell on the MS platform. Our evaluation of this phenomenon using single species samples has highlighted the fact that some barcodes appear to be more prone to this occurrence than others. Also the FG barcoding protocol appears to be more prone to switching. Although the frequency of switching we observed was very low (below 1 %) this could be a major source of error for certain studies where species may be at a high abundance in some samples and absent in other, such as clinical samples being analysed for pathogens.

Analysis of all experimental conditions used (summarised in Fig. 6) demonstrates that amplicon choice has the most pronounced effect on the measurement of the relative abundance of the different species. This is evident in the PB data, which uses the V1 and V9 primers. This primer combination does not detect any of the archaeal species (see Additional file 10: Figure S10 and Additional file 11: Table S1). Also the V1 and V3 primers perform badly in detecting Archaea. Where different platforms are used with the same primers their performance in detecting species is similar. The whole genome shotgun approach gives the most accurate estimation of species abundance in this analysis.

The performance of the different platforms in describing the mock communities can be quantitatively measured by calculating the entropy of the data generated. Our analysis shows that the platforms have a similar performance although the platforms with fewer reads (454 and PB) perform slightly less well. It should also be pointed out that different library methodologies have been used in a previous study to increase base calling accuracy [40] using a mix of primers that have frameshifting nucleotides to increase cluster identification on the Illumina platform. We have also used a mix of primers with random nucleotides for the DI design (data not shown), but ever since the software RTA v1.17.28 release, no significant differences were found. The barcode design (FG or DI) does affect the entropy of the data. In our hands, the DI with V4 primers performed best, giving the highest entropy and therefore lower bias.

It should also be emphasised that although the V4 region appears to be performing the best out of the universal primers tested here it did not capture all the species present, failing to pick up the Chlorobium species, for example. In general, our results confirm those of [28] demonstrating that no single universal primer can capture all the microbial diversity. Therefore primers should be carefully chosen for individual studies based on prior knowledge of the taxa likely to be present or combinations of primers must be used.

Although the relative abundances of species within samples are not well described by 16S sequencing, the ability to estimate the relative abundance of the same species between samples is very good. As demonstrated in Fig. 7, both the PB and MS data correlate well with the different abundances of bacteria in the EM and UM communities. This is despite the small number of reads that are generated by the PB platform.

When classifying at the genera level using the RDP classifier all platforms underestimated the total number of genera, the PB performing the least well. This was again due to the failure of the V1-V9 primers in amplifying the Archaea, as none of these species were present in our dataset.

The findings discussed above were generated by comparing benchmarking datasets against a database of 16S sequences in synthetic communities. However most 16S sequencing studies will use OTU reconstruction to identify species, as in most cases the community structure will not be known. We calculated OTUs from our datasets at the 3 % level. At this granularity our community should have 57 different OTUs, however the PB massively overestimated the number of OTUs despite the low number of reads generated. The MS and IT performed well with the V4 amplicon while the 454 underestimated the number of OTUs. Nevertheless, when the PB data was compared to a database of known OTUs (which we know to be present) it performed better than the other platforms predicting more of the “good OTUs”. So although the MS and IT predict roughly the correct number of OTUs many of these are “noisy OTUs”. In the case of the IT, around 40 % of the predicted OTUs are incorrect.

Rarefaction analysis demonstrates that the short read platforms (IT, MS, 454) Additional file 12: Figure S12 have reached asymptote in most experimental designs. Therefore we would not predict the number of OTUs to increase much with more sequencing. However it is striking how different the OTU estimation is between different experimental designs and replicates. However in the case of the PB, the rarefaction curves appear linear, other than the CCS8 reads. This demonstrates that the CCS sequencing can improve the data substantially but the result of this will be a reduction in the total number of reads.

## Conclusion

We have used synthetic microbial communities for this and our previous study [28] and while our communities are relatively complex compared to many other published studies they are not going to be representative of most environmental samples, which are likely to have much more species diversity across a wider range of abundances. Here, we demonstrate that consistency in input DNA quantity, PCR cycles and barcoding strategy is required to get reproducible and comparable results. The best design of the experiment will depend on the questions asked of the data and what prior knowledge exists. For example, we have shown that if the species present in a sample are known then PacBio is better than other platforms for identification and therefore it can be used for confirmation studies, conversely if OTU estimation is done blind then the short read platforms perform best. There has recently been a heightened awareness about the over-interpretation of microbiome studies [17]. We hope that this analysis will better inform future experimental designs and interpretation but also highlight areas where out technology can be improved to better represent microbial diversity.

## Availability of supporting data

The datasets are available on the European Nucleotide Archive under the study accession number: ERP005737 (http://www.ebi.ac.uk/ena/data/view/ERP005737).

## Additional files

Additional file 1
**Figure S1.** AMPLImock pipeline. (PDF 886 kb)

Additional file 2
**Figure S2.** Read quality distribution for PacBio. (PDF 5 kb)

Additional file 3
**Figure S3.** Error Transition Probabilities for all platforms. (ZIP 34 kb)

Additional file 4
**Figure S4.** Impact of platform and region on entropy. (ZIP 16 kb)

Additional file 5
**Figure S5.** Impact of library preparation method on barcode switching probability. (ZIP 16 kb)

Additional file 6
**Figure S6.** Heatmap of observed species in the single-species libraries. (PDF 32 kb)

Additional file 7
**Figure S7.** Taxonomic profiling of different platforms. (PDF 7 kb)

Additional file 8
**Figure S8.** OTU comparison for all platforms. (PDF 8 kb)

Additional file 9
**Figure S9.** Impact of PCR cycles on OTUs. (ZIP 43 kb)

Additional file 10
**Figure S10.** Heatmap of arc heal species in the EM community. (ZIP 9 kb)

Additional file 11
**Figure S1.** Regression of abundances against primer mismatches and 16S rRNA gene true copy numbers. (PDF 50 kb)

Additional file 12
**Table S12.** Rarefaction curves for all platforms. (PDF 107 kb)
