# Supplementary material for: A comprehensive benchmarking study of protocols and sequencing platforms for 16S rRNA community profiling
Source: BMC Genomics. 2016 Jan 14;17:55. doi: 10.1186/s12864-015-2194-9 (PMC4712552; doi:10.1186/s12864-015-2194-9)
Supplement: Additional file 1 — Figure S1. AMPLImock pipeline. (PDF 886 kb) [file 12864_2015_2194_MOESM1_ESM.pdf]

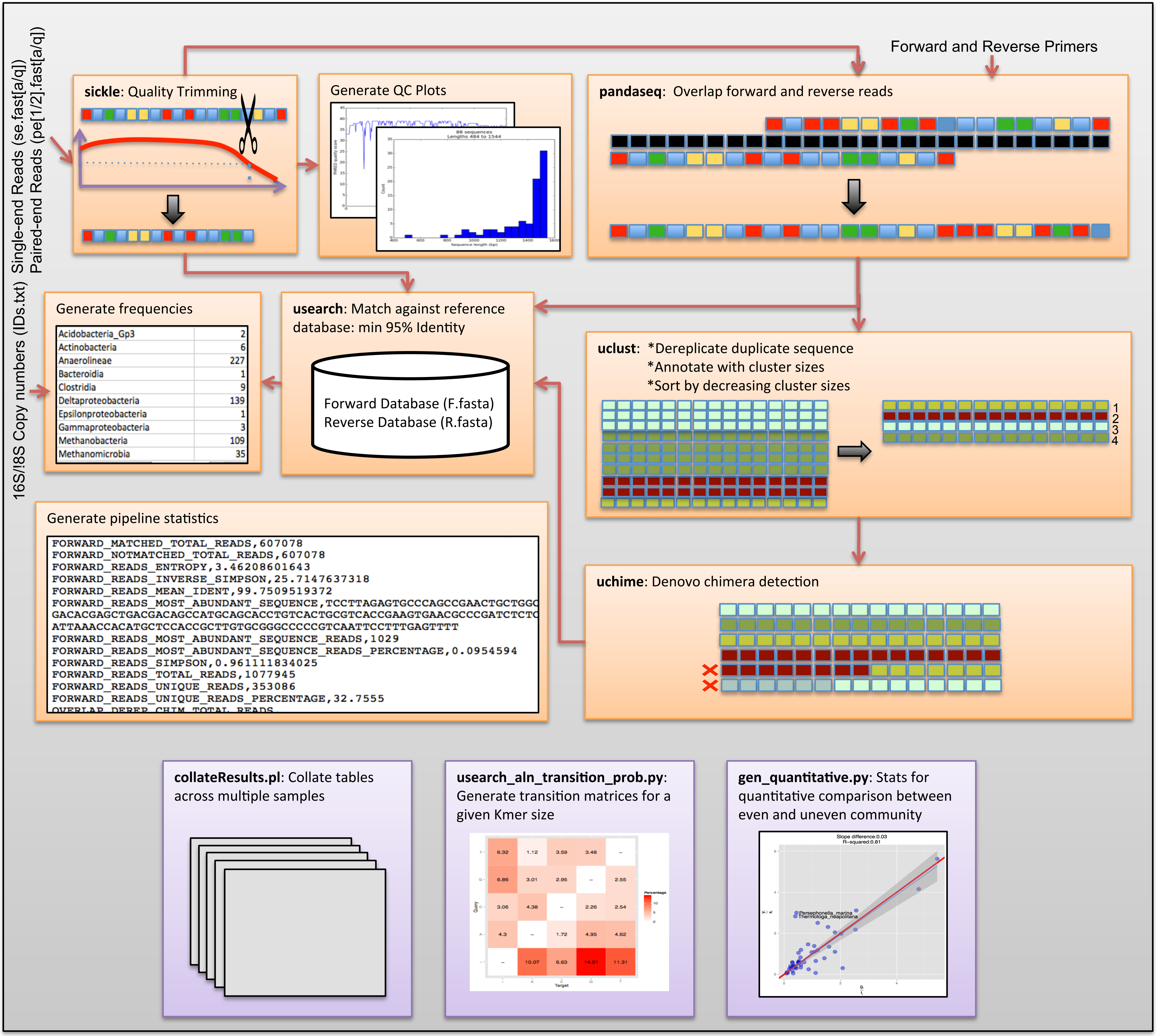

Single-end Reads (se.fast[a/q])
Paired-end Reads (pe[1/2].fast[a/q])
16S/!8S Copy numbers (IDs.txt)
Forward and Reverse Primers
sickle: Quality Trimming
Generate QC Plots
88 sequences
Lengths 484 to 1544
Sequence length (bp)
pandaseq: Overlap forward and reverse reads
Generate frequencies
Acidobacteria_Gp3 2
Actinobacteria 6
Anaerolineae 227
Bacteroidia 1
Clostridia 9
Deltaproteobacteria 139
Epsilonproteobacteria 1
Gammaproteobacteria 3
Methanobacteria 109
Methanomicrobia 35
usearch: Match against reference database: min 95% Identity
Forward Database (F.fasta)
Reverse Database (R.fasta)
uclust: *Dereplicate duplicate sequence
*Annotate with cluster sizes
*Sort by decreasing cluster sizes
1
2
3
4
Generate pipeline statistics
FORWARD_MATCHED_TOTAL_READS,607078
FORWARD_NOTMATCHED_TOTAL_READS,607078
FORWARD_READS_ENTROPY,3.46208601643
FORWARD_READS_INVERSE_SIMPSON,25.7147637318
FORWARD_READS_MEAN_IDENT,99.7509519372
FORWARD_READS_MOST_ABUNDANT_SEQUENCE,TCCTTAGAGTGCCCAGCCGAACTGCTGGC
GACACGAGCTGACGACAGCCATGCAGCACCTGTCACTGCGTCACCGAAGTGAACGCCCGATCTCTC
ATTAAACCACATGCTCCACCGCTTGTGCGGGCCCCCGTCAATTCCTTTGAGTTT
FORWARD_READS_MOST_ABUNDANT_SEQUENCE_READS,1029
FORWARD_READS_MOST_ABUNDANT_SEQUENCE_READS_PERCENTAGE,0.0954594
FORWARD_READS_SIMPSON,0.961111834025
FORWARD_READS_TOTAL_READS,1077945
FORWARD_READS_UNIQUE_READS,353086
FORWARD_READS_UNIQUE_READS_PERCENTAGE,32.7555
uchime: Denovo chimera detection
collateResults.pl: Collate tables across multiple samples
usearch_aln_transition_prob.py: Generate transition matrices for a given Kmer size
6.32 1.12 3.59 3.48 –
6.86 3.01 2.95 – 2.55
3.06 4.38 – 2.26 2.54
4.3 – 1.72 4.95 4.62
– 10.07 6.63 14.01 11.31
Percentage
Query
Target
gen_quantitative.py: Stats for quantitative comparison between even and uneven community
Slope difference:0.03
R-squared:0.81
Persephonella_marina
Thermotoga_neapolitana
