# Supplementary figures and images for: A comprehensive benchmarking study of protocols and sequencing platforms for 16S rRNA community profiling
Source: BMC Genomics. 2016 Jan 14;17:55. doi: 10.1186/s12864-015-2194-9 (PMC4712552; doi:10.1186/s12864-015-2194-9)

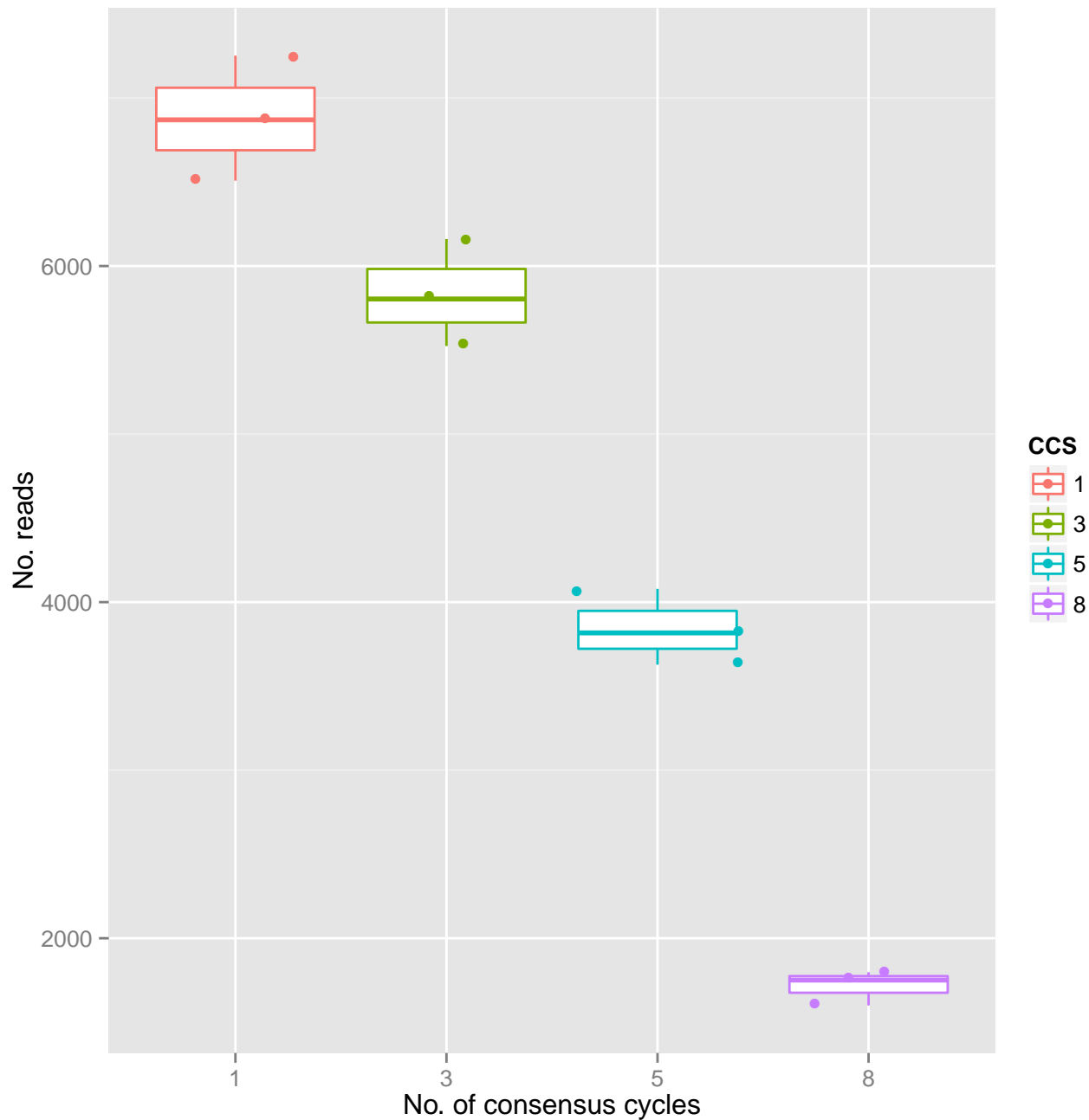

Supplement: Additional file 2 — Figure S2. Read quality distribution for PacBio. (PDF 5 kb) [file 12864_2015_2194_MOESM2_ESM.pdf]

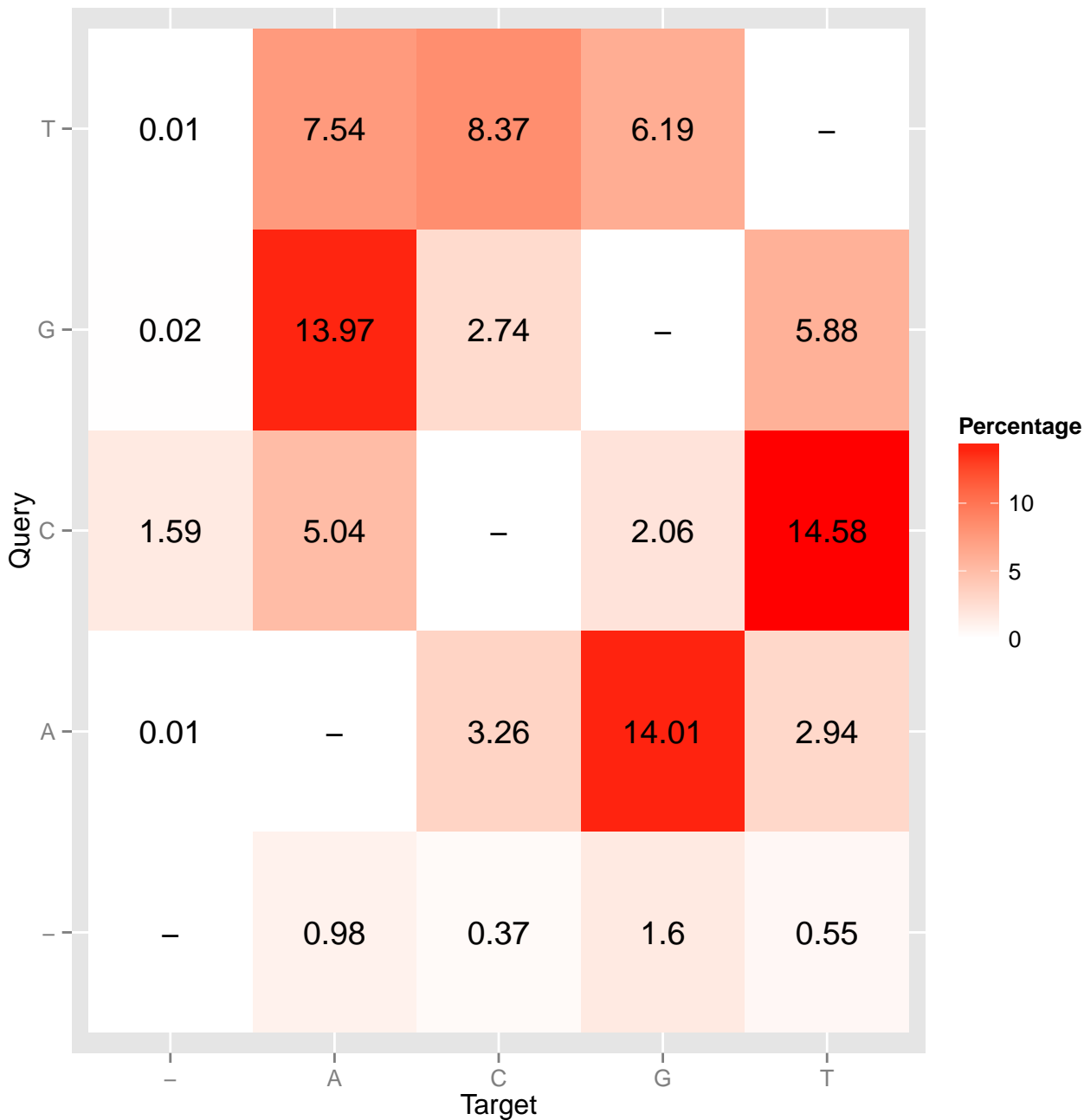

Supplement: Additional file 3 — Figure S3. Error Transition Probabilities for all platforms. (ZIP 34 kb) [file 12864_2015_2194_MOESM3_ESM.zip › FigureS3a.pdf]

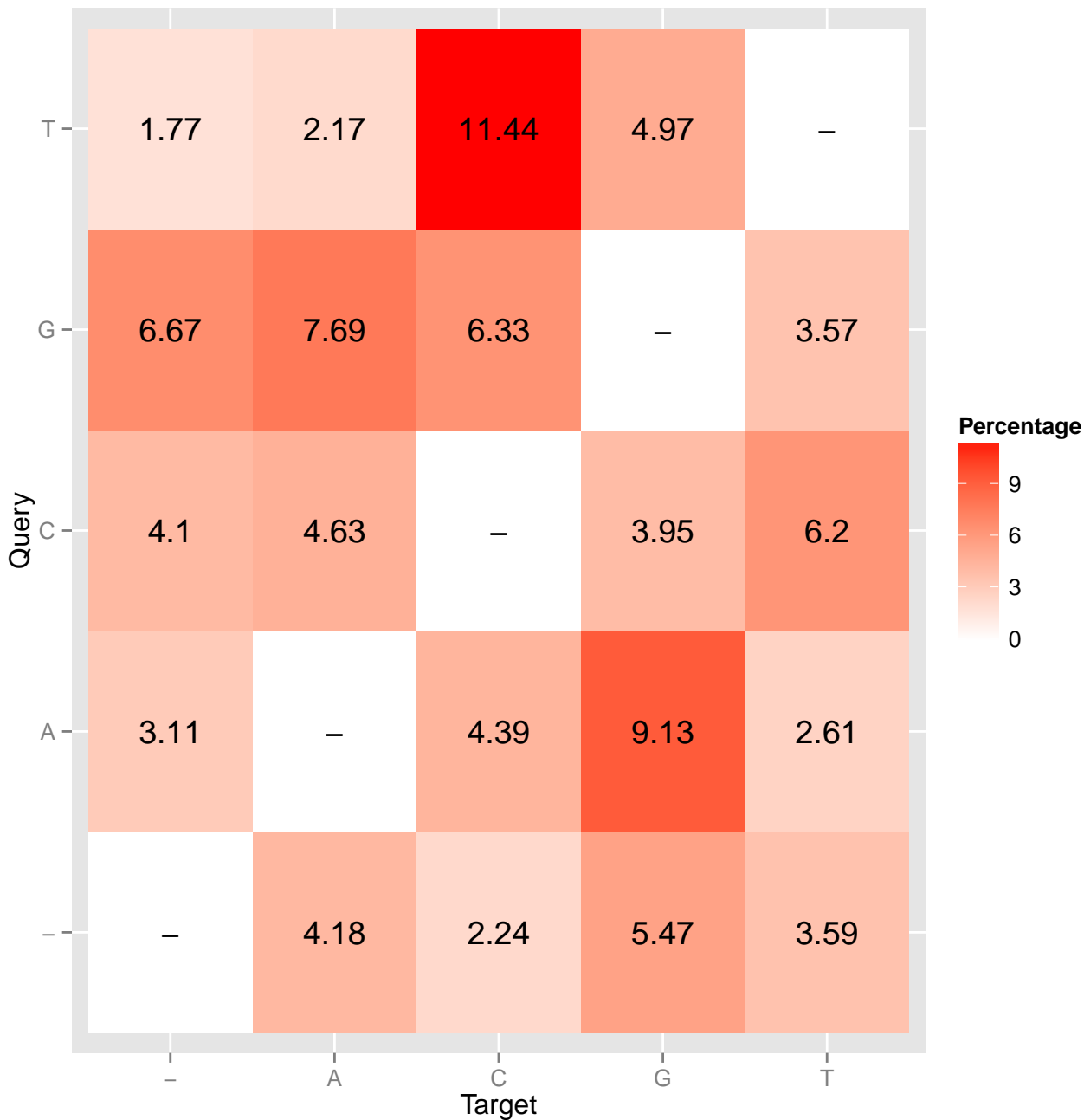

Supplement: Additional file 3 — Figure S3. Error Transition Probabilities for all platforms. (ZIP 34 kb) [file 12864_2015_2194_MOESM3_ESM.zip › FigureS3b.pdf]

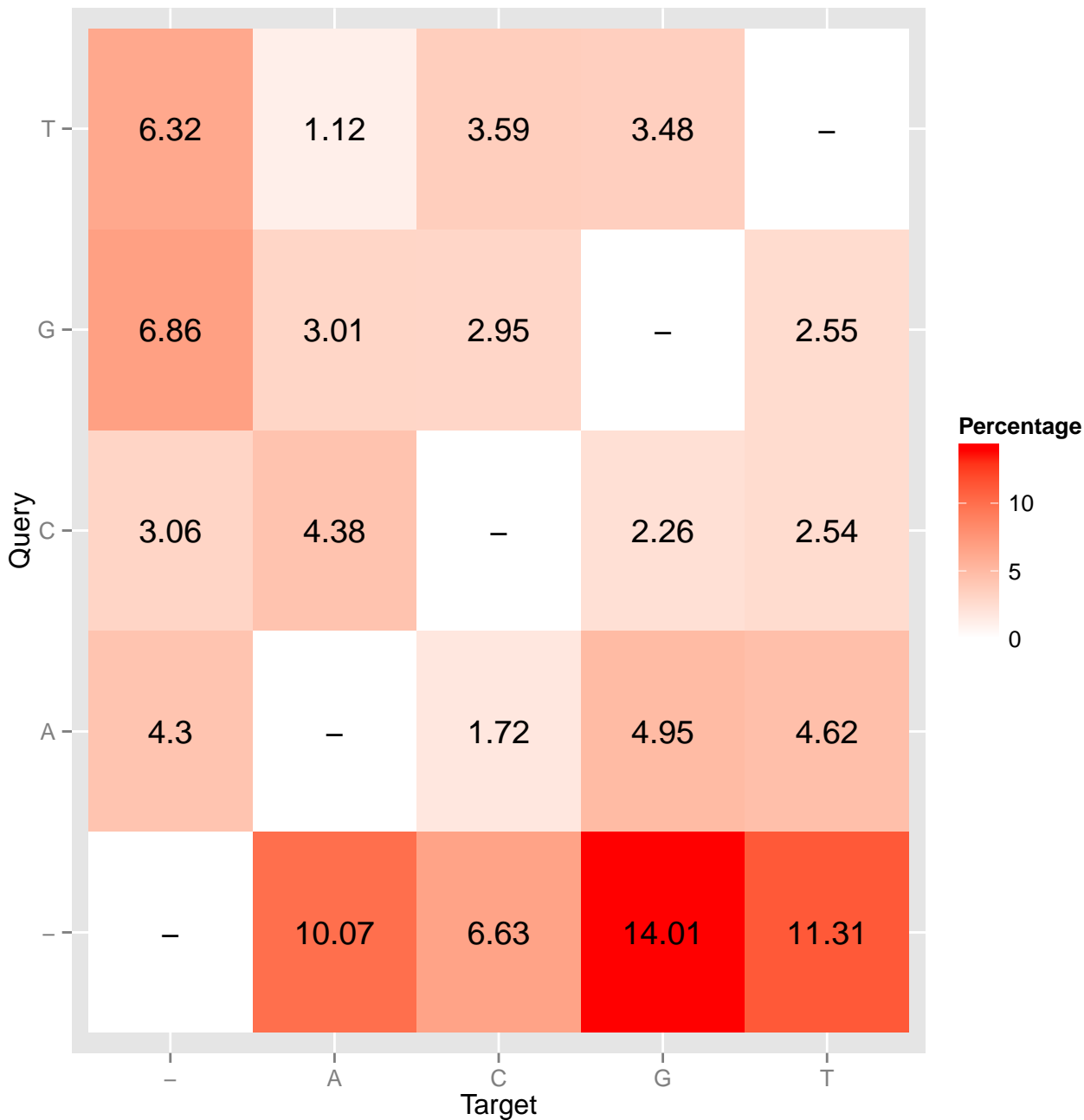

Supplement: Additional file 3 — Figure S3. Error Transition Probabilities for all platforms. (ZIP 34 kb) [file 12864_2015_2194_MOESM3_ESM.zip › FigureS3c.pdf]

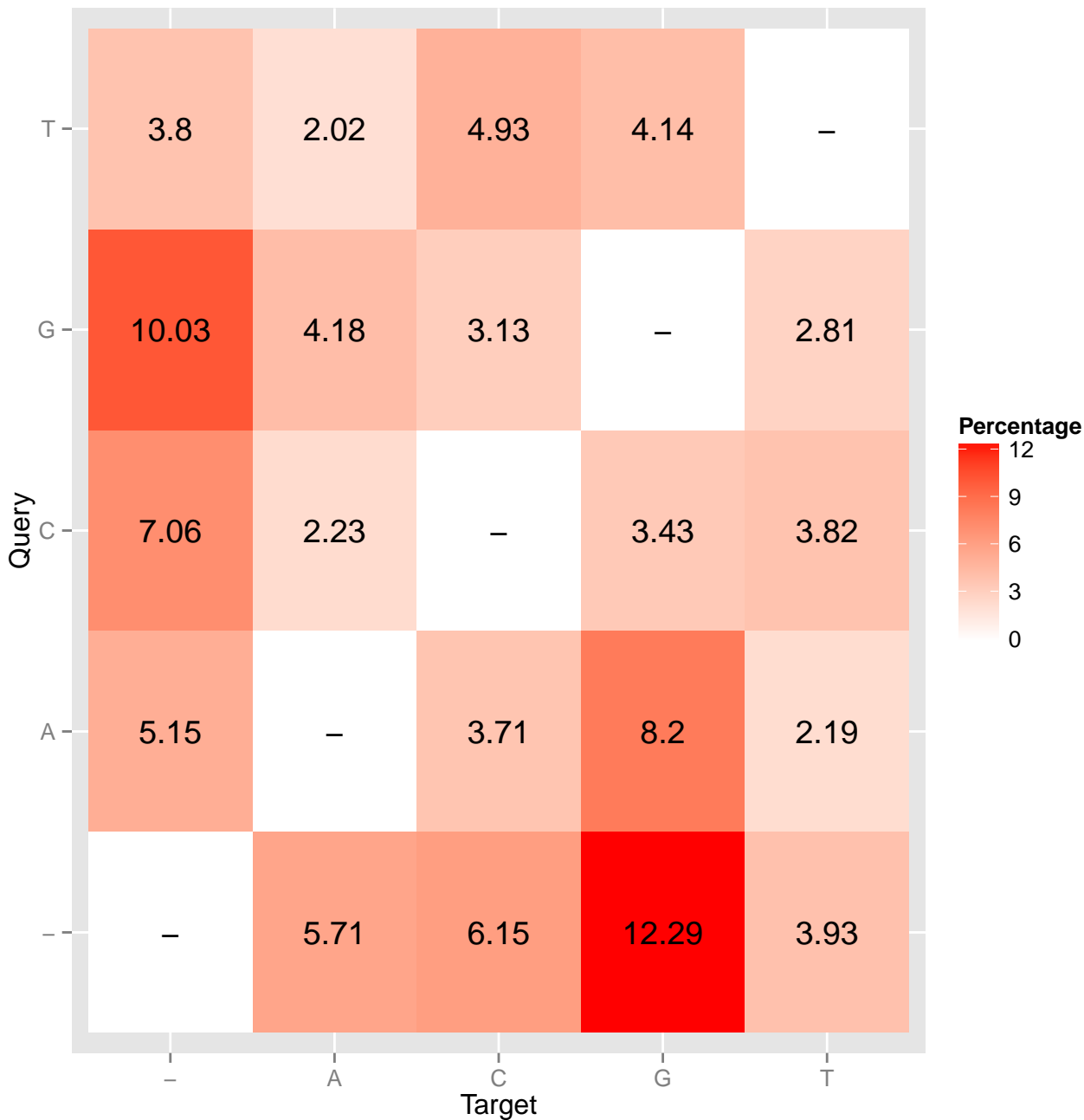

Supplement: Additional file 3 — Figure S3. Error Transition Probabilities for all platforms. (ZIP 34 kb) [file 12864_2015_2194_MOESM3_ESM.zip › FigureS3d.pdf]

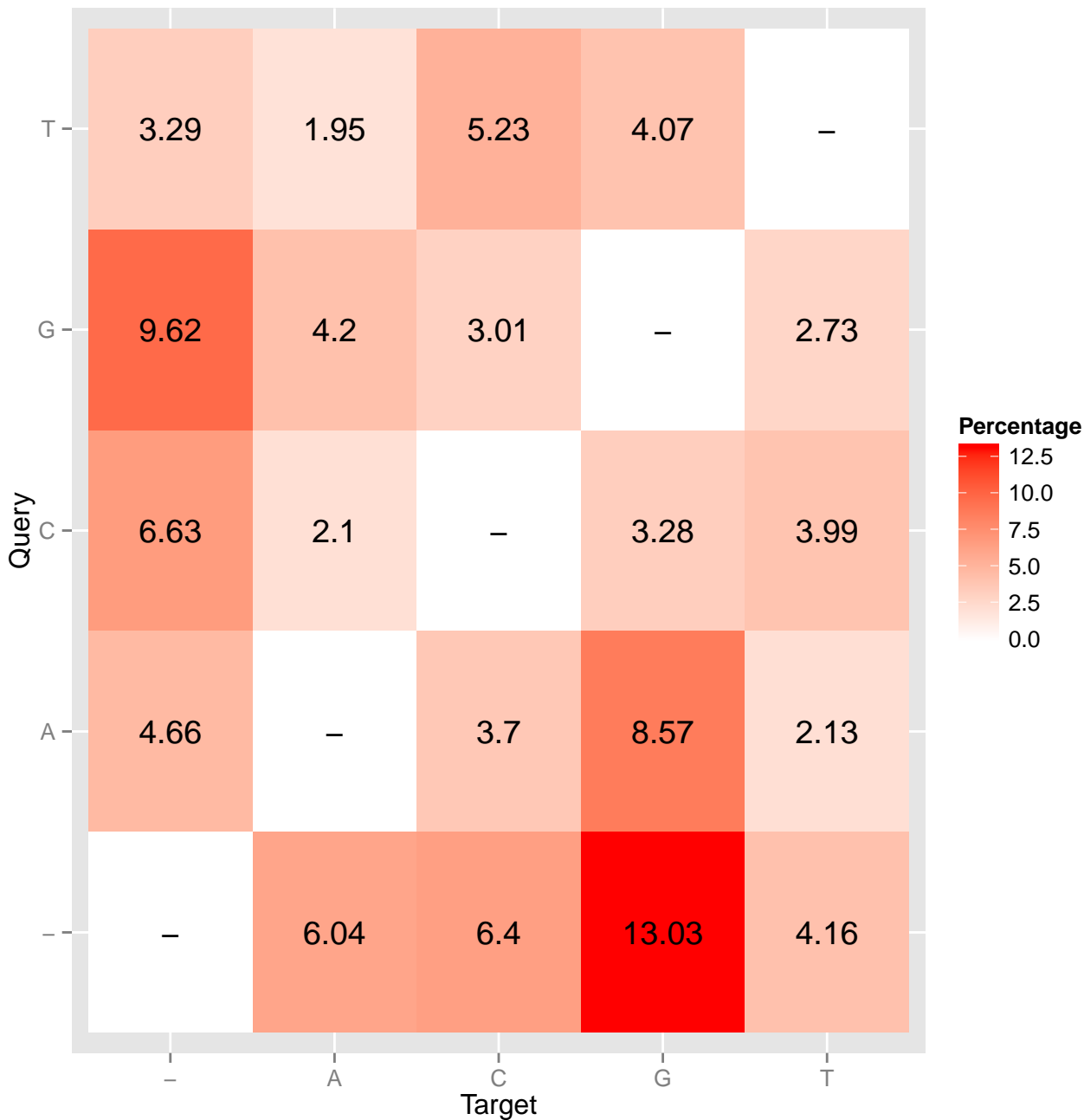

Supplement: Additional file 3 — Figure S3. Error Transition Probabilities for all platforms. (ZIP 34 kb) [file 12864_2015_2194_MOESM3_ESM.zip › FigureS3e.pdf]

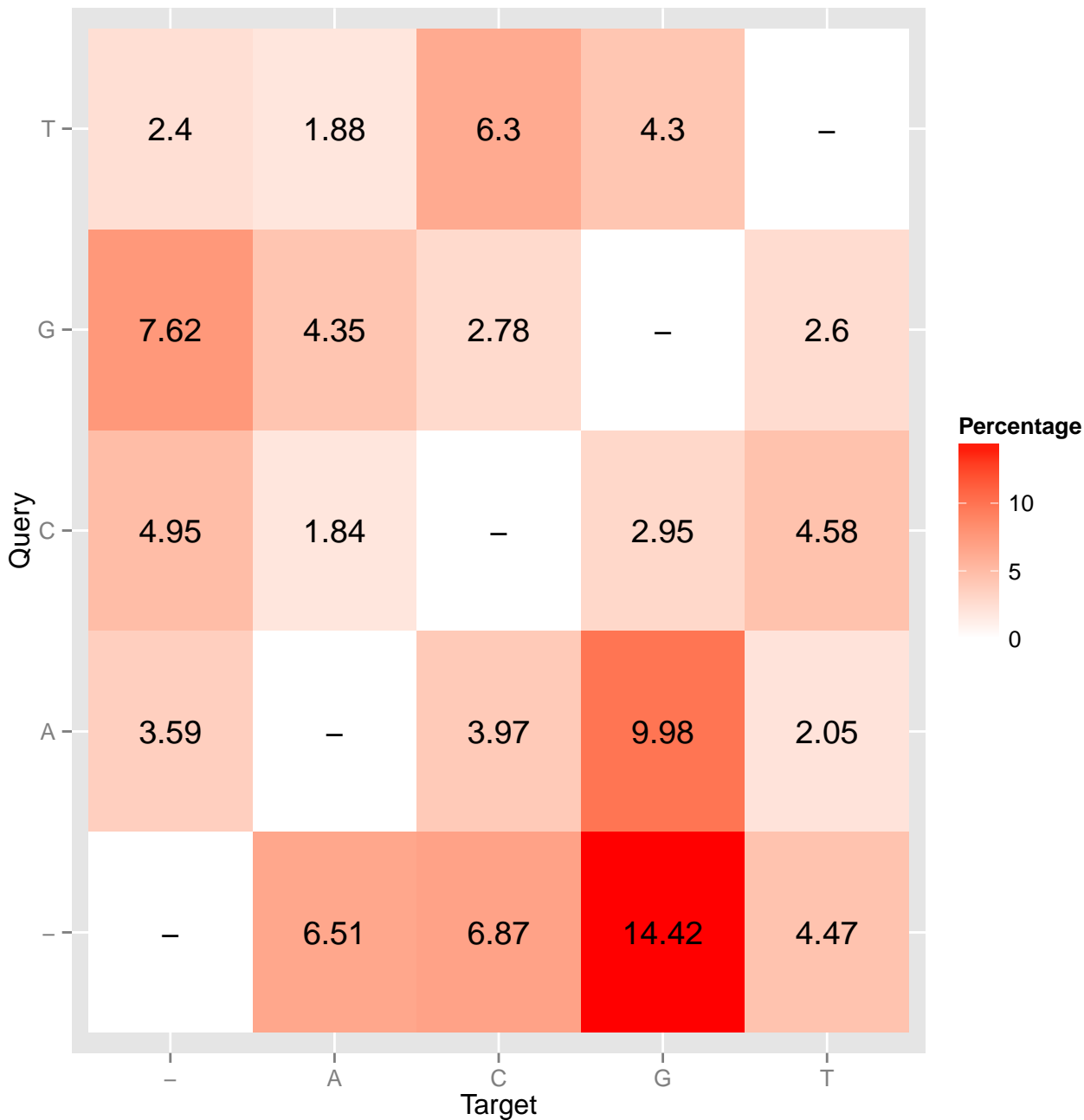

Supplement: Additional file 3 — Figure S3. Error Transition Probabilities for all platforms. (ZIP 34 kb) [file 12864_2015_2194_MOESM3_ESM.zip › FigureS3f.pdf]

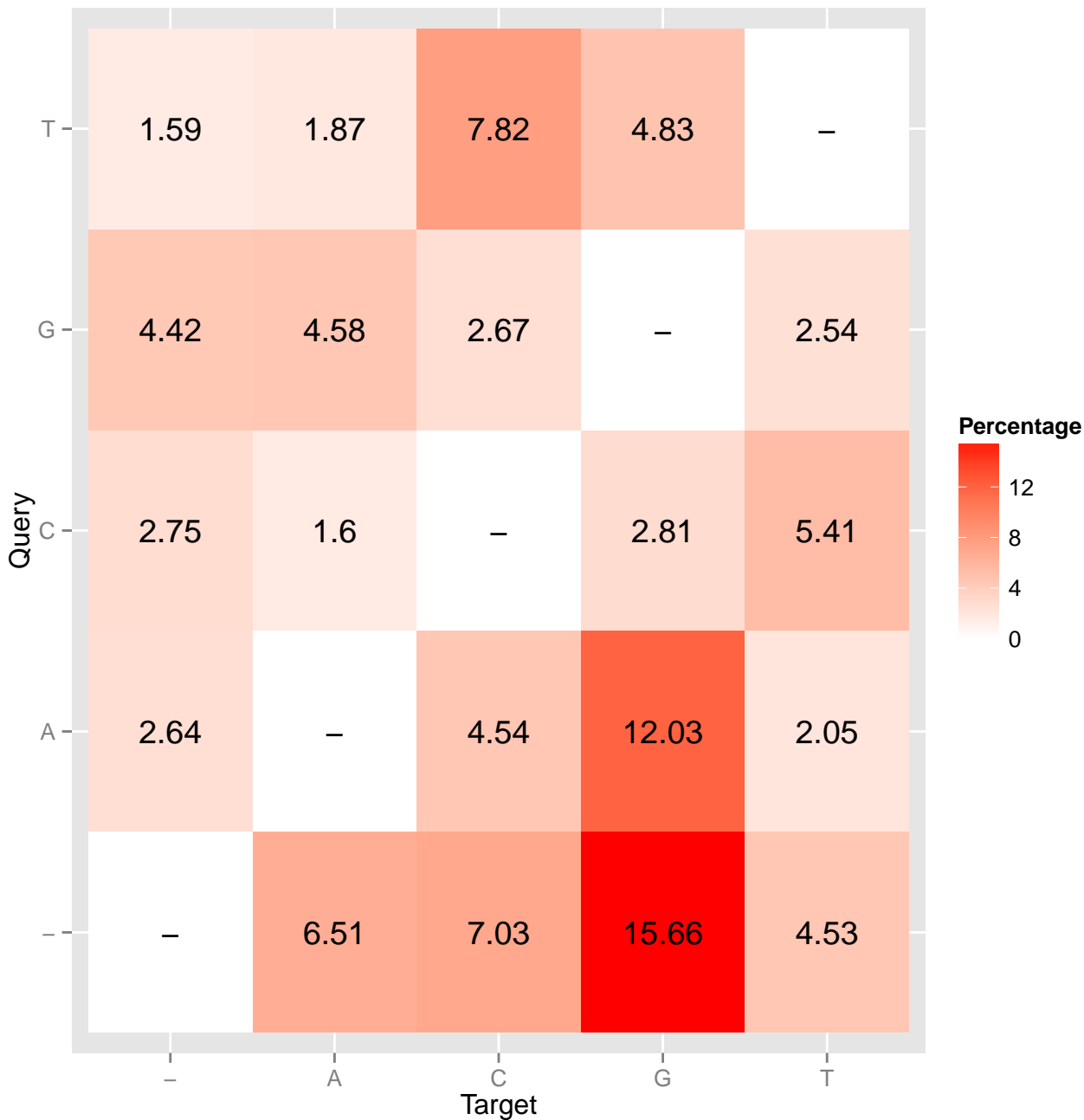

Supplement: Additional file 3 — Figure S3. Error Transition Probabilities for all platforms. (ZIP 34 kb) [file 12864_2015_2194_MOESM3_ESM.zip › FigureS3g.pdf]

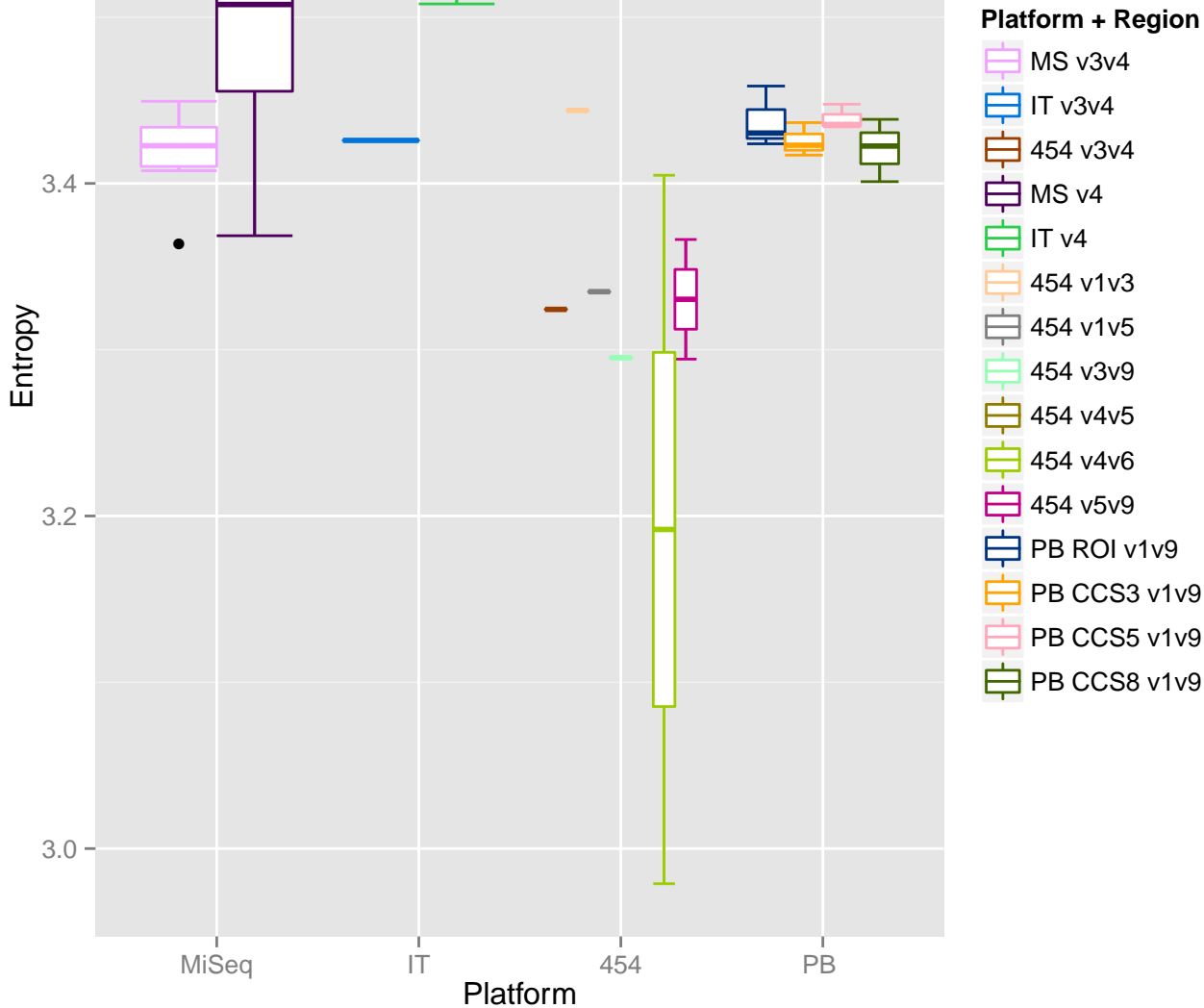

Supplement: Additional file 4 — Figure S4. Impact of platform and region on entropy. (ZIP 16 kb) [file 12864_2015_2194_MOESM4_ESM.zip › FigureS4a.pdf]

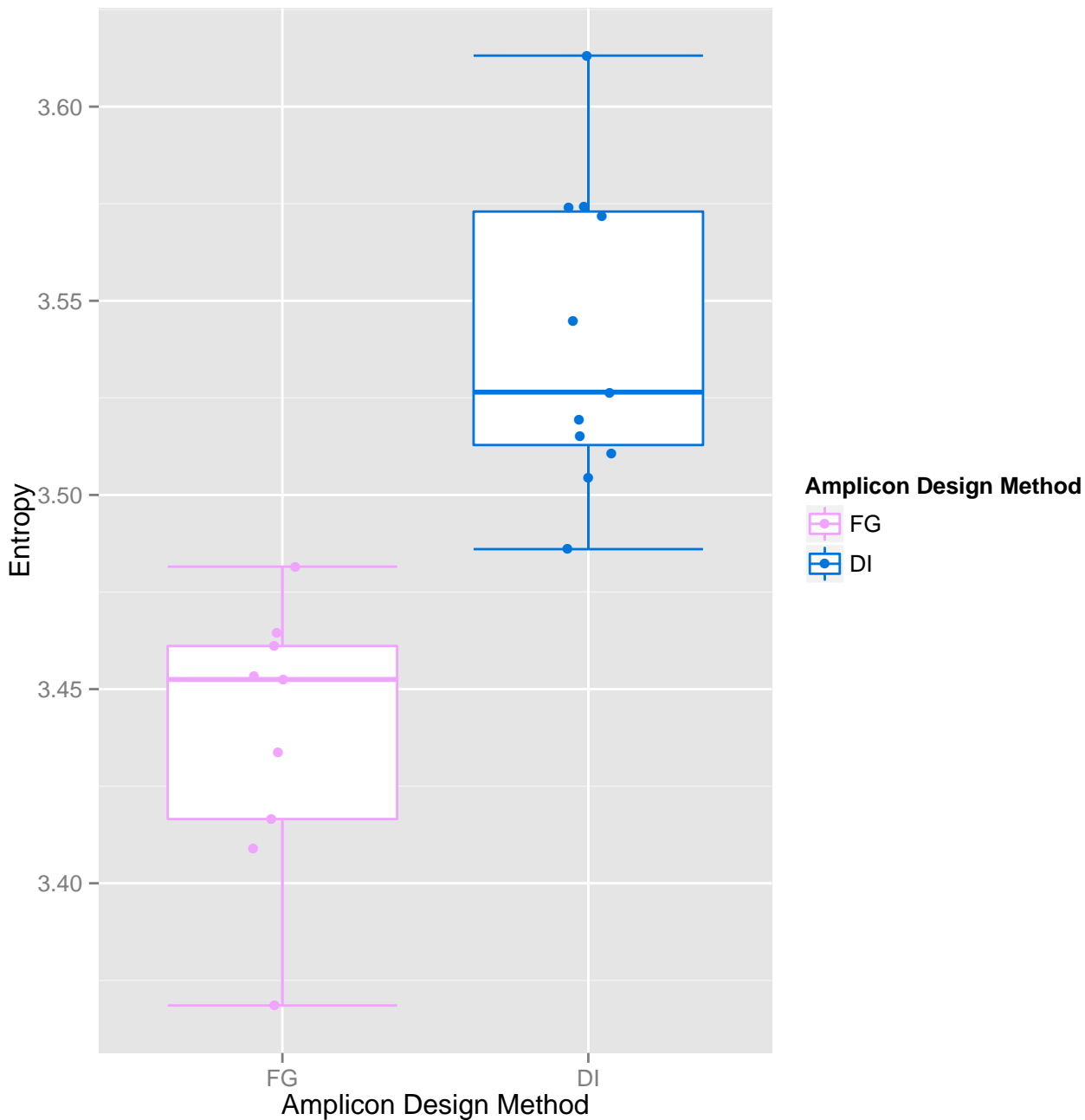

Supplement: Additional file 4 — Figure S4. Impact of platform and region on entropy. (ZIP 16 kb) [file 12864_2015_2194_MOESM4_ESM.zip › FigureS4b.pdf]

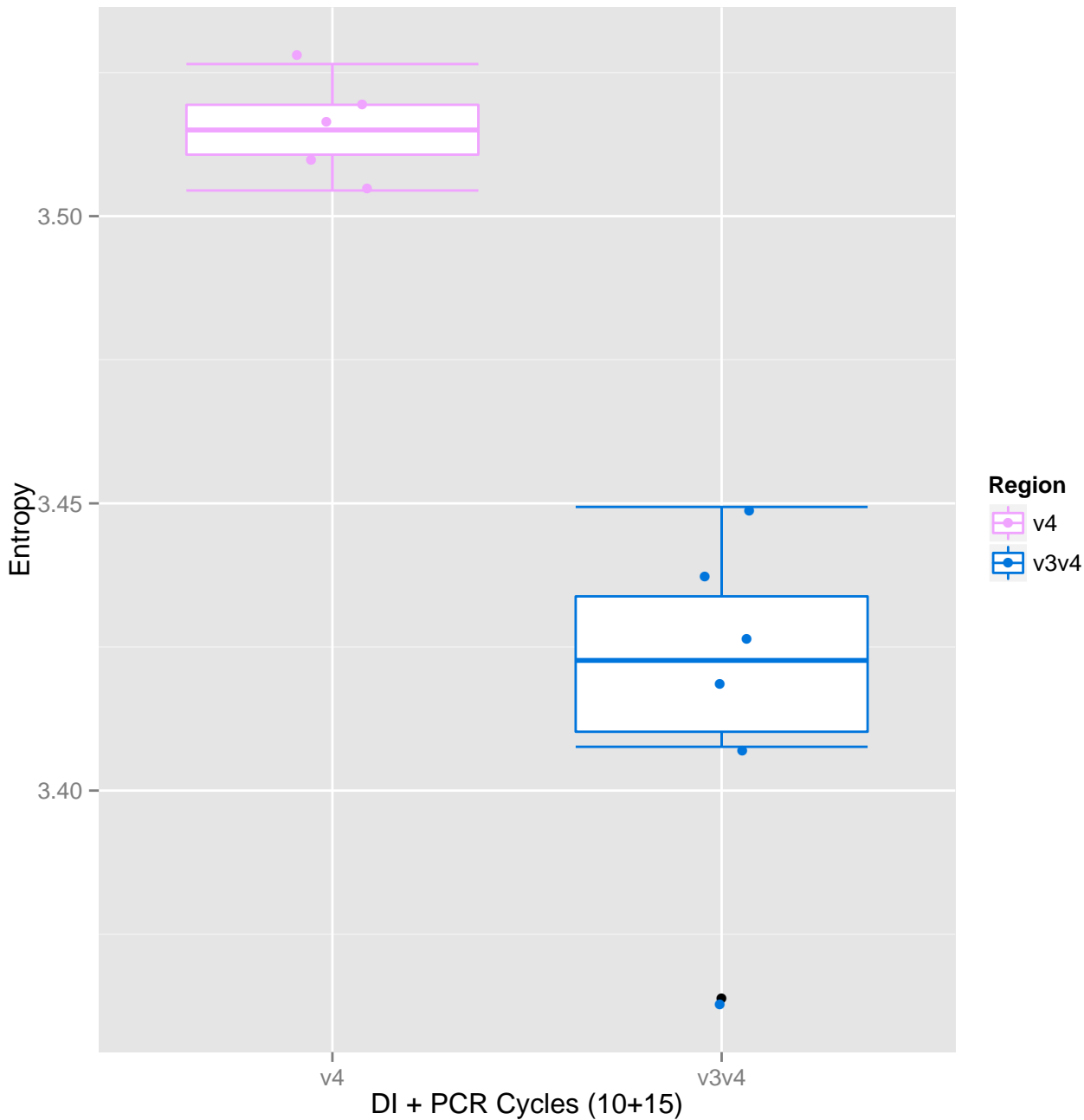

Supplement: Additional file 4 — Figure S4. Impact of platform and region on entropy. (ZIP 16 kb) [file 12864_2015_2194_MOESM4_ESM.zip › FigureS4c.pdf]

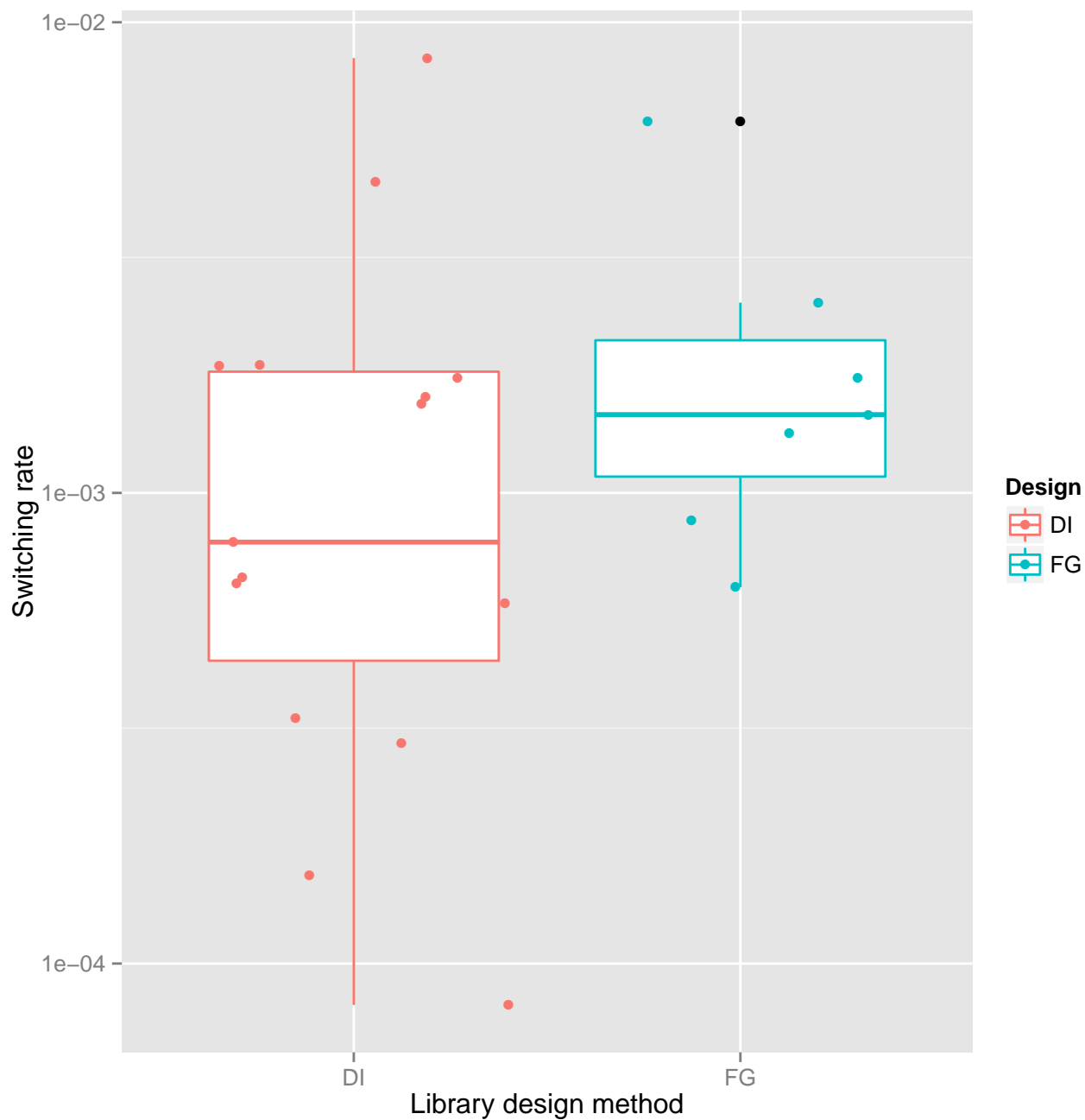

Supplement: Additional file 5 — Figure S5. Impact of library preparation method on barcode switching probability. (ZIP 16 kb) [file 12864_2015_2194_MOESM5_ESM.pdf]

# Color Key and Histogram

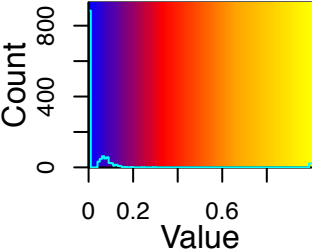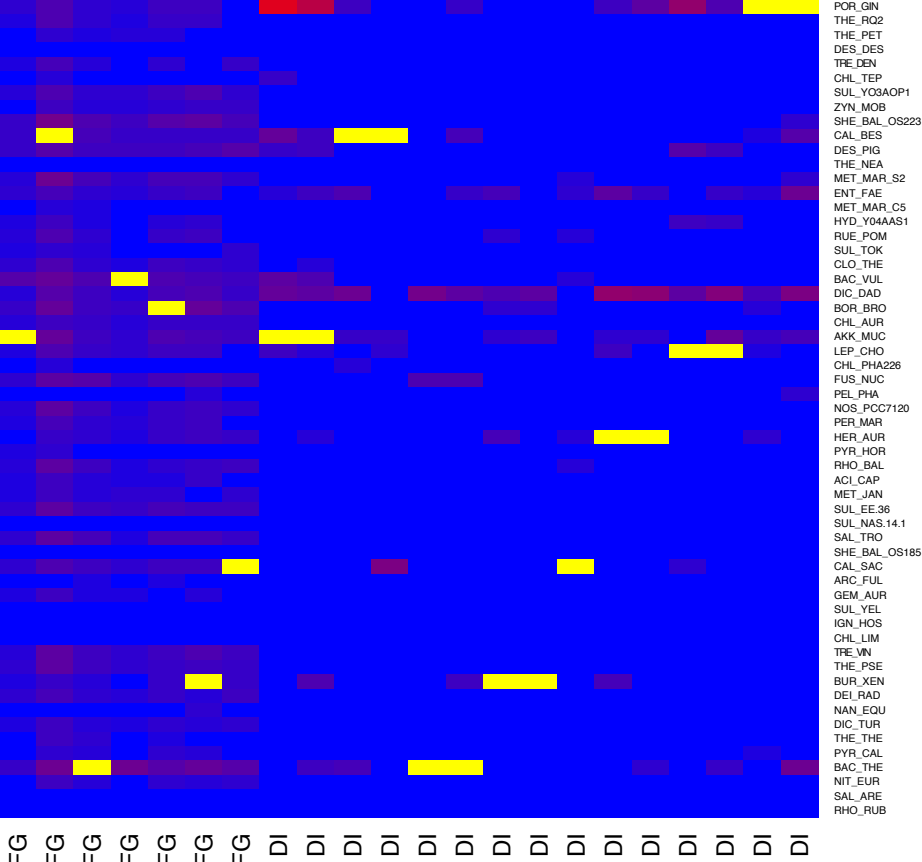

Supplement: Additional file 6 — Figure S6. Heatmap of observed species in the single-species libraries. (PDF 32 kb) [file 12864_2015_2194_MOESM6_ESM.pdf]

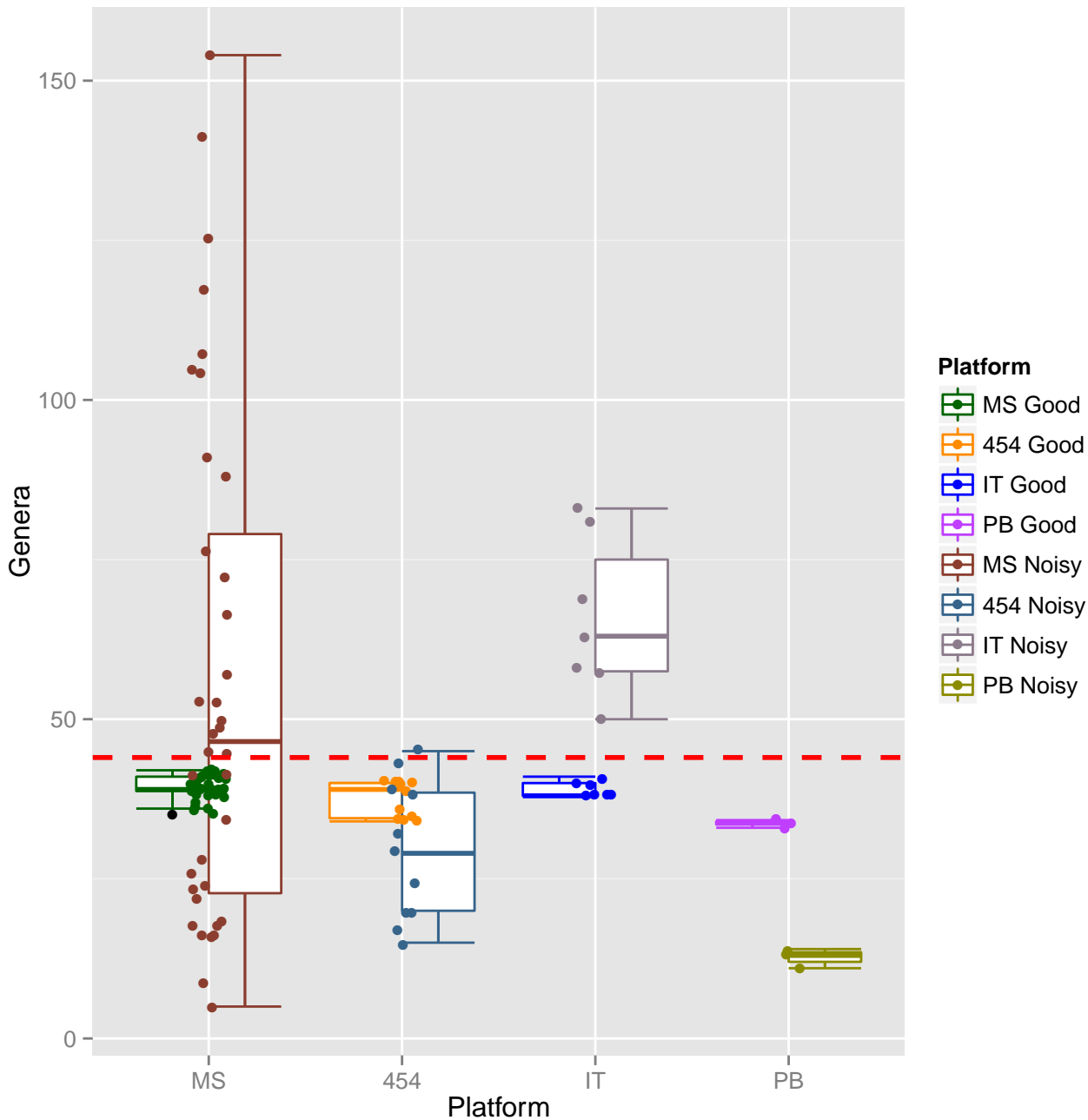

Supplement: Additional file 7 — Figure S7. Taxonomic profiling of different platforms. (PDF 7 kb) [file 12864_2015_2194_MOESM7_ESM.pdf]

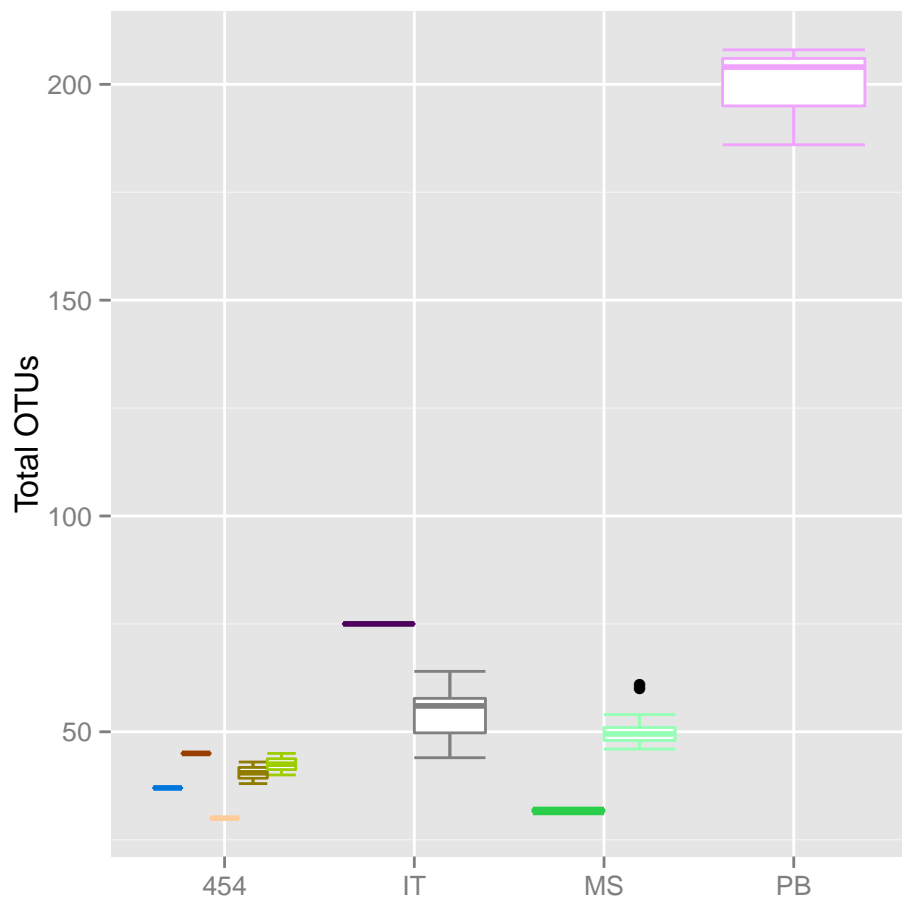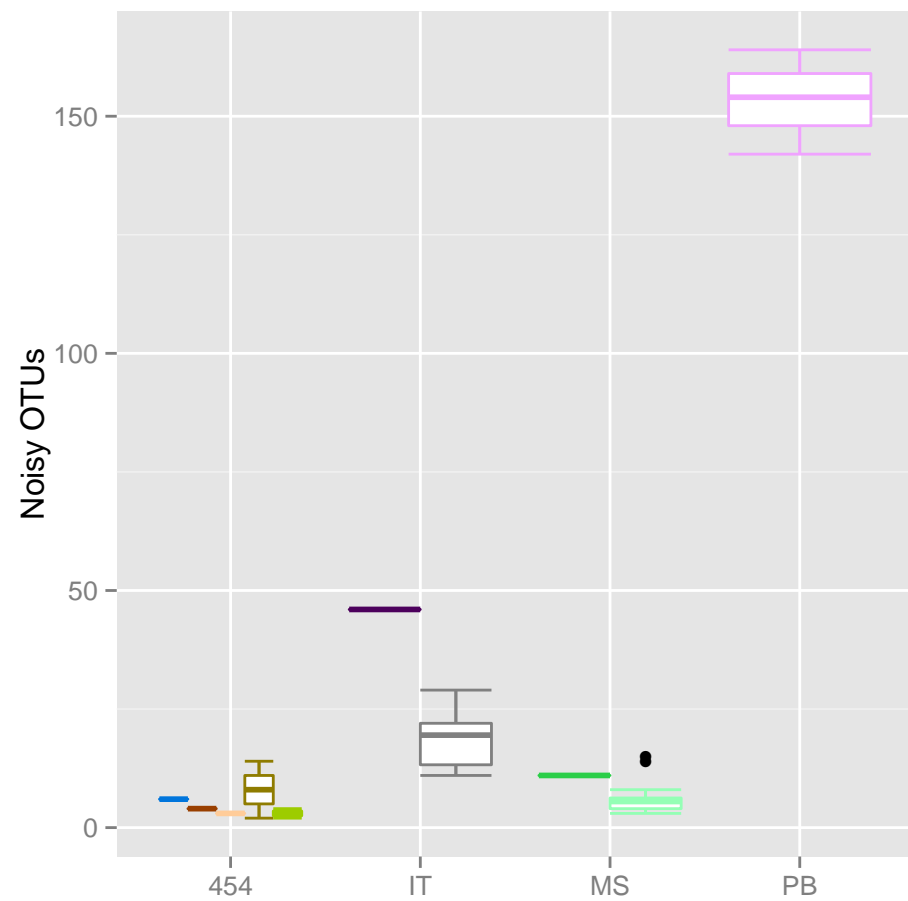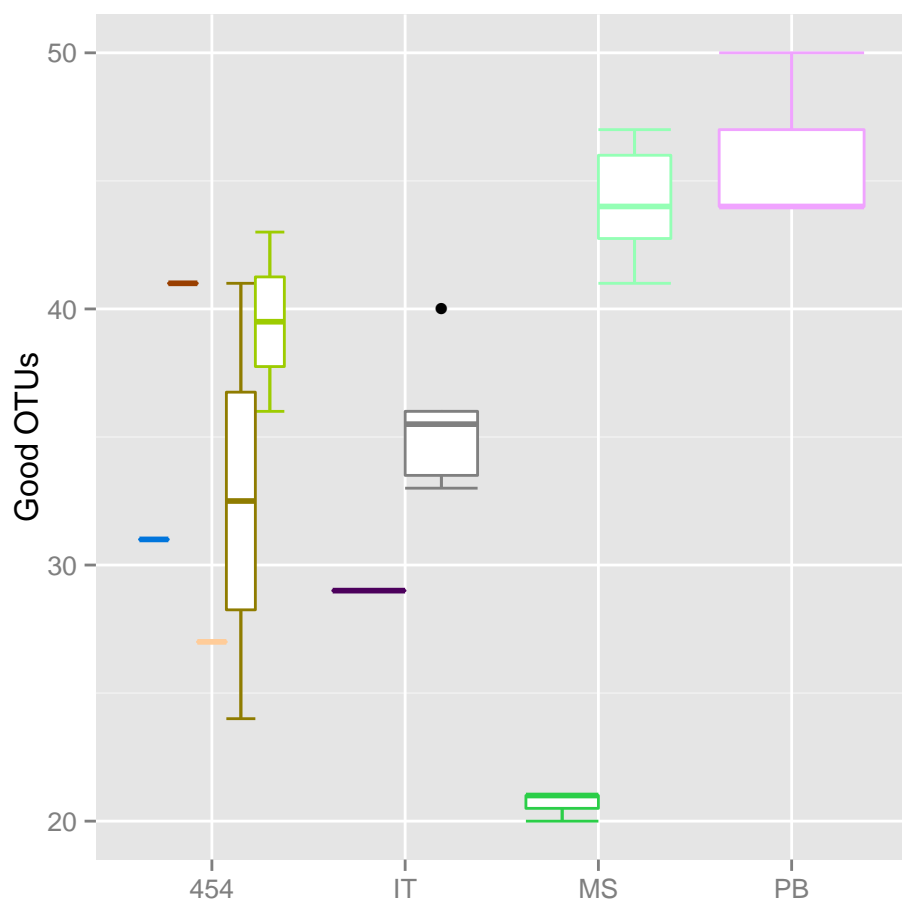

**Platform + Region**

- PB CCS8 v1v9
- 454 v1v5
- 454 v3v4
- IT v3v4
- MS v3v4
- 454 v3v9
- IT v4
- MS v4
- 454 v4v6
- 454 v5v9

Supplement: Additional file 8 — Figure S8. OTU comparison for all platforms. (PDF 8 kb) [file 12864_2015_2194_MOESM8_ESM.pdf]

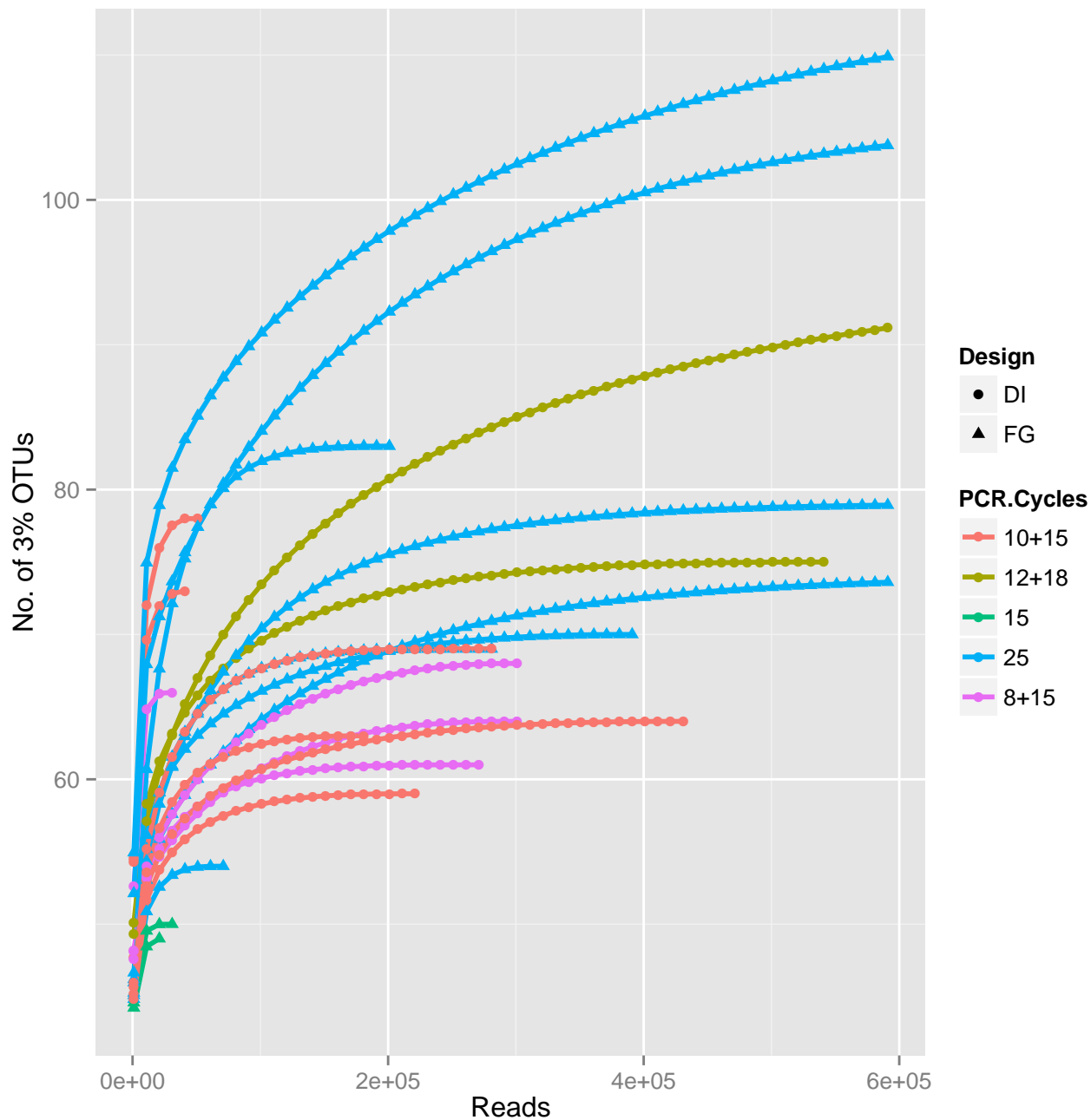

Supplement: Additional file 9 — Figure S9. Impact of PCR cycles on OTUs. (ZIP 43 kb) [file 12864_2015_2194_MOESM9_ESM.zip › FigureS9a.pdf]

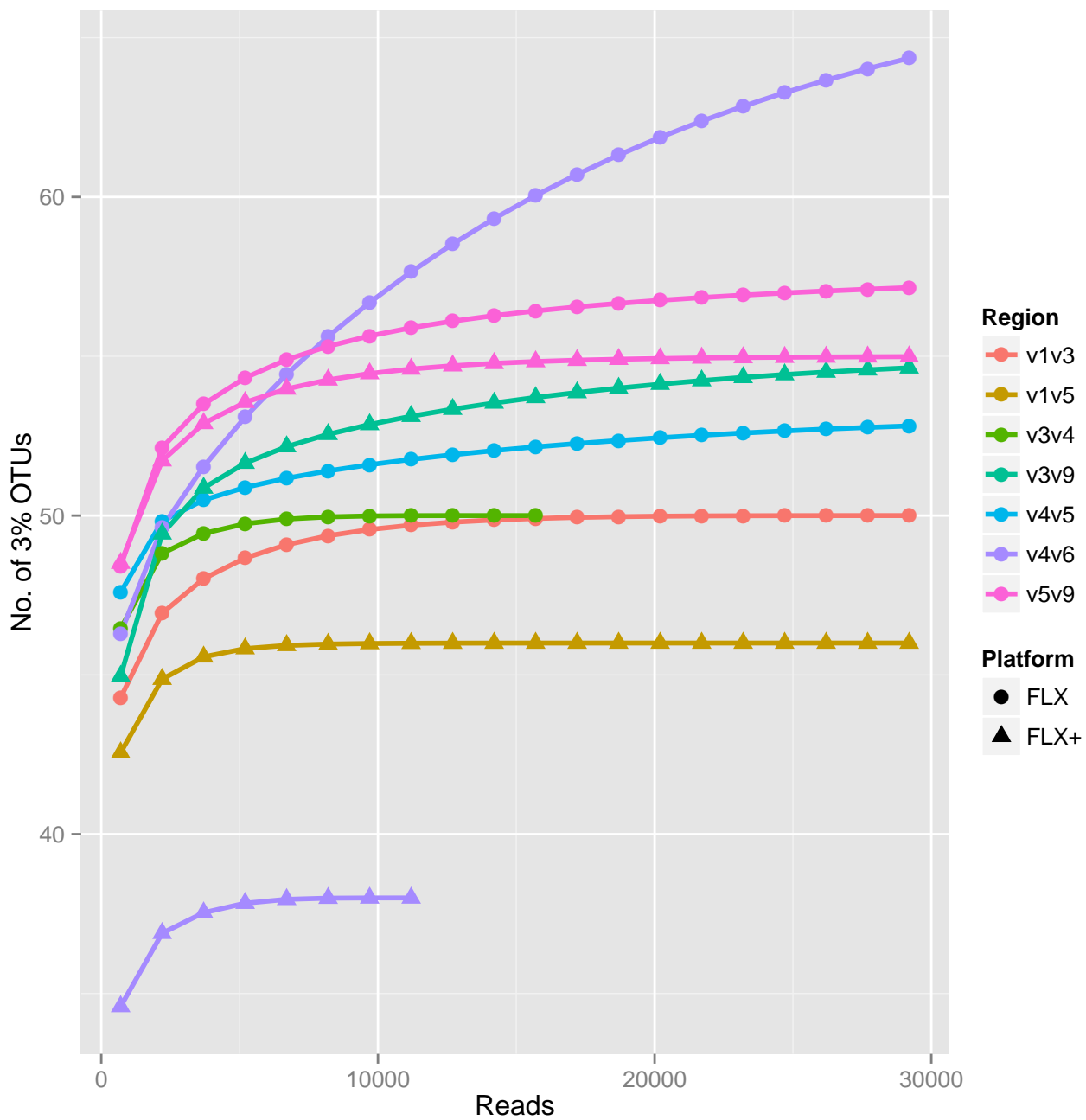

Supplement: Additional file 9 — Figure S9. Impact of PCR cycles on OTUs. (ZIP 43 kb) [file 12864_2015_2194_MOESM9_ESM.zip › FigureS9b.pdf]

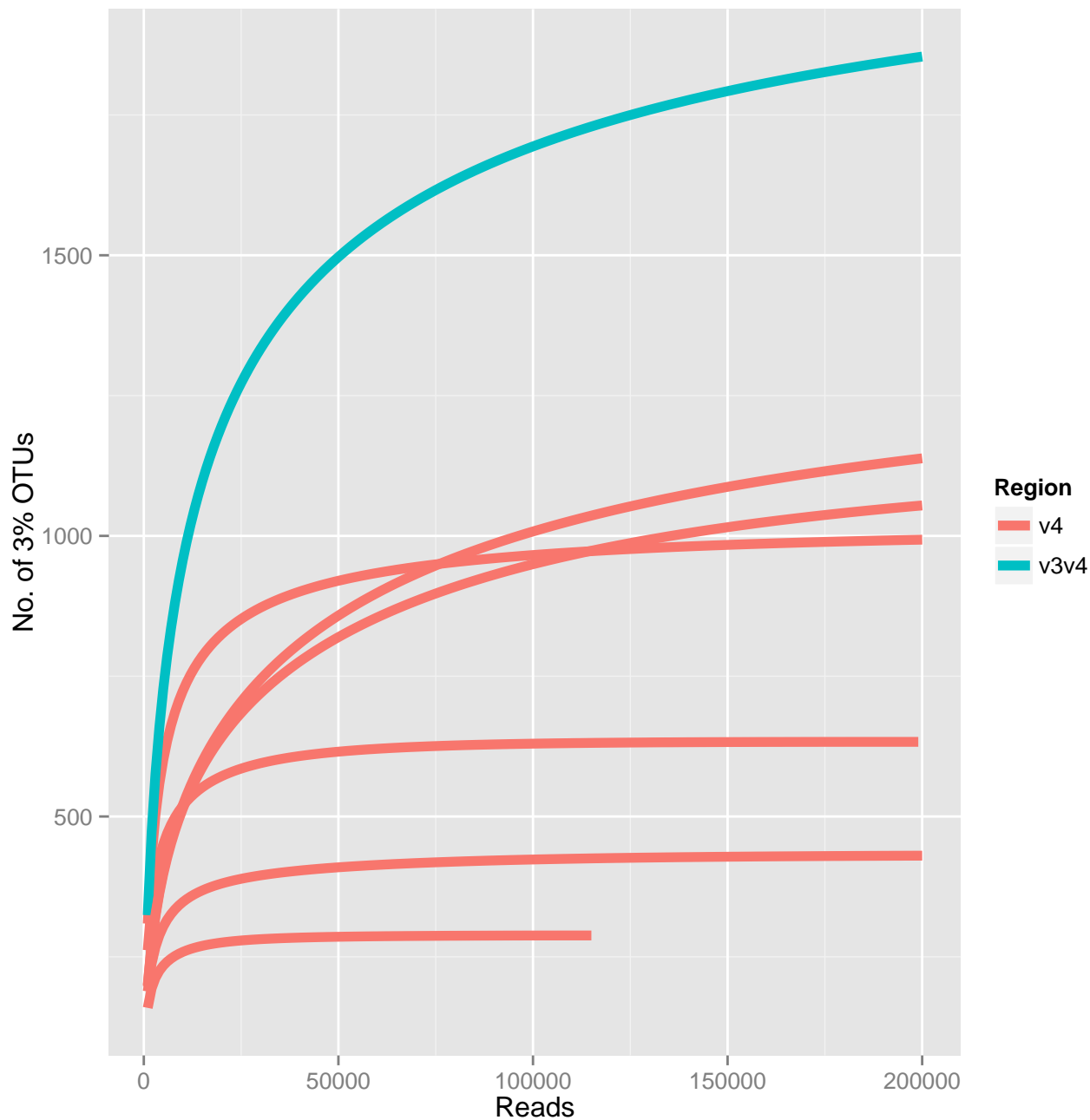

Supplement: Additional file 9 — Figure S9. Impact of PCR cycles on OTUs. (ZIP 43 kb) [file 12864_2015_2194_MOESM9_ESM.zip › FigureS9c.pdf]

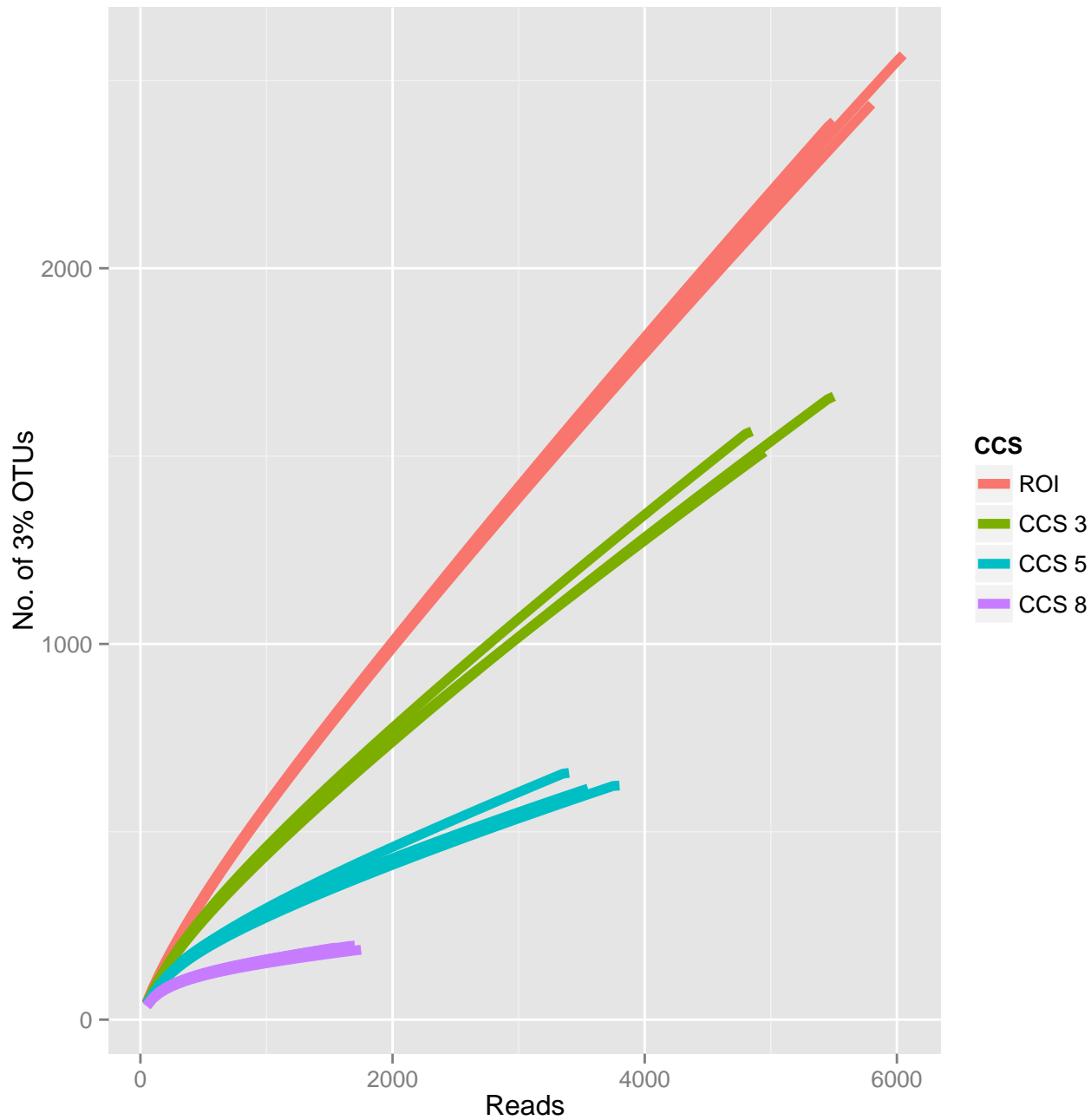

Supplement: Additional file 9 — Figure S9. Impact of PCR cycles on OTUs. (ZIP 43 kb) [file 12864_2015_2194_MOESM9_ESM.zip › FigureS9d.pdf]

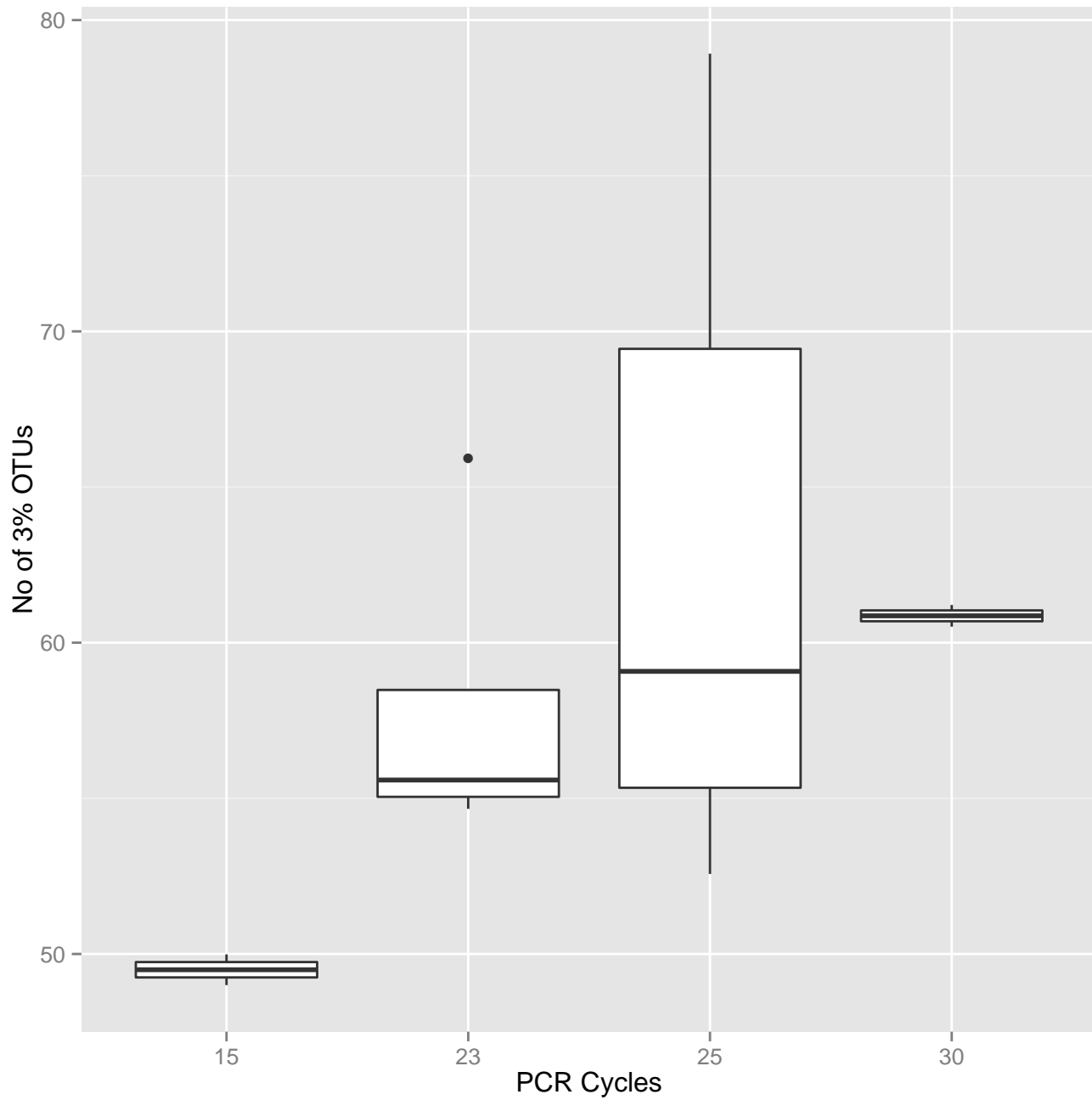

Supplement: Additional file 10 — Figure S10. Heatmap of arc heal species in the EM community. (ZIP 9 kb) [file 12864_2015_2194_MOESM10_ESM.zip › FigureS10a.pdf]

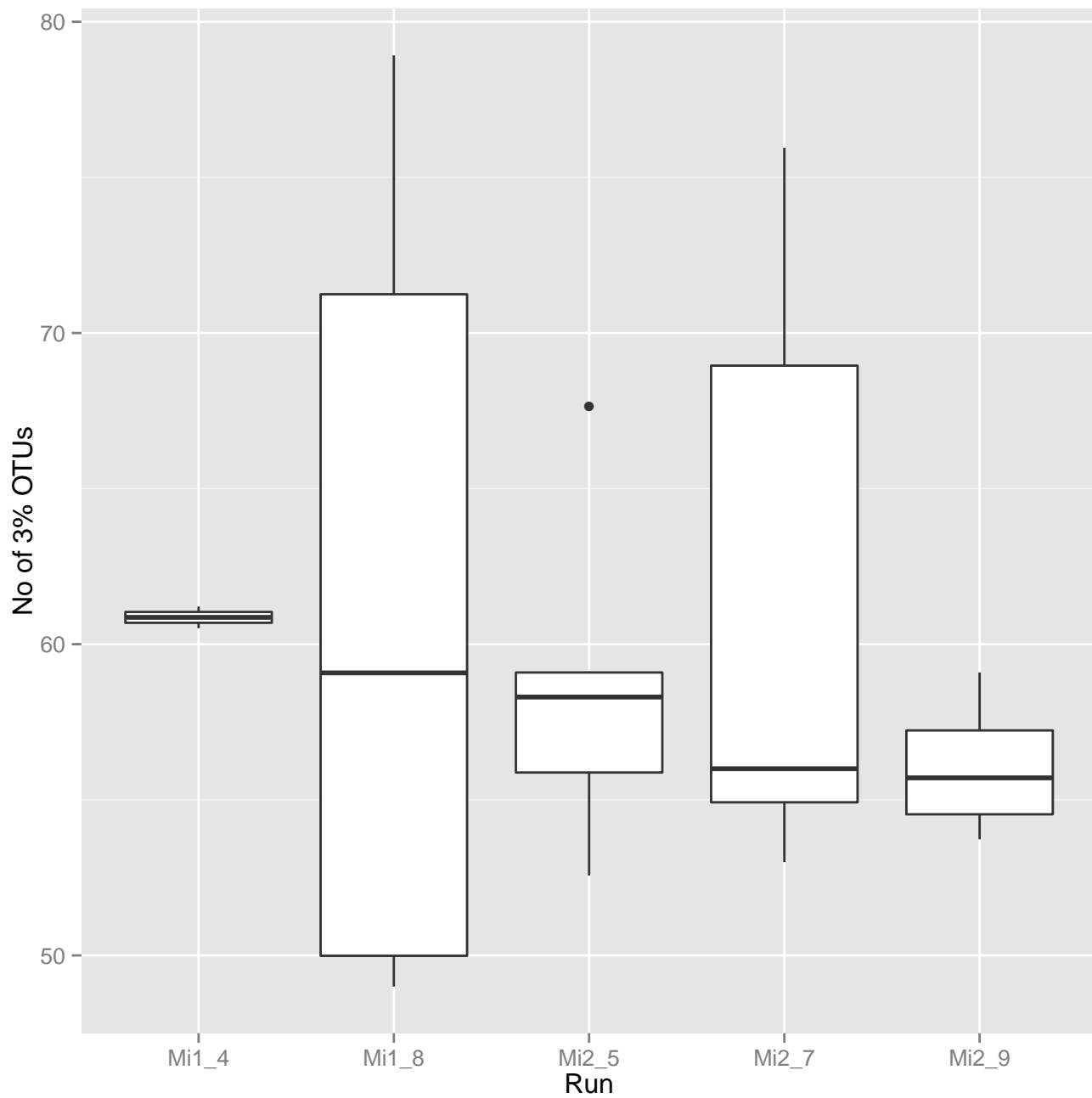

Supplement: Additional file 10 — Figure S10. Heatmap of arc heal species in the EM community. (ZIP 9 kb) [file 12864_2015_2194_MOESM10_ESM.zip › FigureS10b.pdf]

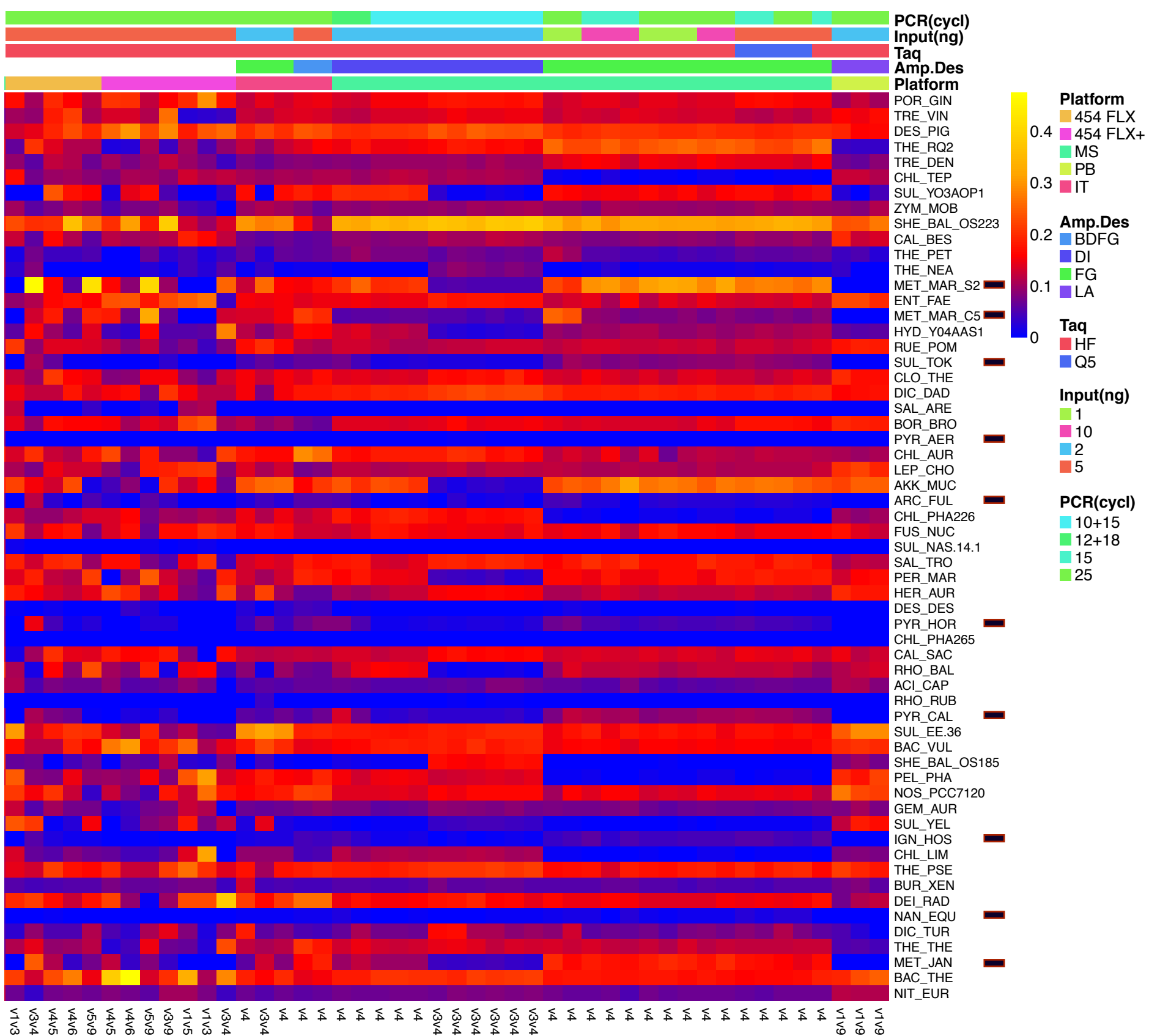

Supplement: Additional file 11 — Figure S1. Regression of abundances against primer mismatches and 16S rRNA gene true copy numbers. (PDF 50 kb) [file 12864_2015_2194_MOESM11_ESM.pdf]
