# Supplementary material for: A comprehensive benchmarking study of protocols and sequencing platforms for 16S rRNA community profiling
Source: BMC Genomics. 2016 Jan 14;17:55. doi: 10.1186/s12864-015-2194-9 (PMC4712552; doi:10.1186/s12864-015-2194-9)
Supplement: Additional file 12 — Table S12. Rarefaction curves for all platforms. (PDF 107 kb) [file 12864_2015_2194_MOESM12_ESM.pdf]

Table S1: **Regression of abundances against primer mismatches and 16S rRNA gene true copy numbers**. The results are shown for EM community samples from MS ($n = 26$), 454 ($n = 12$), IT ($n = 3$), and PB ($n = 3$). We considered the community members ($n = 53$) for which true gene copy numbers were found in the ribosomal RNA operons database `https://rrndb.umms.med.umich.edu/`. For each sample, the primers (Table 3) were aligned against the reference sequences ($n = 128$) and number of mismatches were calculated. Where multiple sequences were available in the reference database, the number of mismatches were averaged. We then fitted a linear regression model $log(abundance) = \beta_0 + \beta_{Mismatches} Mismatches + \beta_{Copy\ numbers} Copy\ numbers$ using `lm()` in `R` for each platform and primer (by collating the data from all those samples where the specific primer was considered). Note that the β estimates along with p values and significances are shown.

Platform	Primer ID	β_0	$\beta_{Mismatches}$	$\beta_{Copy\ numbers}$
MS	FG515for	-6.4368 [7.5787e-88 ***]	-1.6042 [8.8015e-15 ***]	0.0914 [0.2337]
MS	1Round515For	-6.3996 [7.9462e-39 ***]	-1.6908 [2.639e-08 ***]	0.25127 [0.024899 *]
MS	1Round341For	-7.8809 [1.2078e-61 ***]	0.9794 [0.098092 .]	0.59721 [1.4648e-08 ***]
MS	FG8xxrev	-7.828 [1.3978e-86 ***]	0.091548 [0.035449 *]	0.28859 [0.00048314 ***]
MS	1Round805RARev	-7.1849 [1.1231e-34 ***]	1.1782 [0.073244 .]	0.3009 [0.023811 *]
MS	1Round806R	-7.4326 [1.3695e-51 ***]	3.1576 [0.028476 *]	0.29938 [0.0092547 **]
IT	454_F515A	-6.4176 [3.2798e-18 ***]	0.91934 [0.26518]	0.2307 [0.16687]
IT	TtP1_Kn805rev	-6.0102 [1.7795e-30 ***]	-1.5194 [5.1285e-07 ***]	0.24347 [0.031585 *]
IT	454_F341	-6.0963 [8.4806e-16 ***]	-1.2067 [0.0017117 **]	0.3355 [0.021154 *]
454	454_27YMF	-9.9159 [1.5865e-20 ***]	-0.17606 [0.45883]	0.87658 [0.0001302 ***]
454	454_515R	-10.38 [1.4911e-17 ***]	1.2783 [0.3376]	0.89089 [0.0014375 **]
454	454_F515	-8.6734 [1.8522e-31 ***]	1.0439 [0.207]	0.58199 [0.00069222 ***]
454	454_926R	-8.6321 [1.079e-20 ***]	1.3573 [0.19984]	0.48266 [0.02683 *]
454	454_1061R	-9.65 [2.1966e-18 ***]	0.15235 [0.51209]	0.88997 [9.0238e-05 ***]
454	454_F787	-6.6957 [2.4895e-20 ***]	-1.3222 [0.13064]	0.36341 [0.020924 *]
454	454_1492R	-7.2682 [3.8237e-29 ***]	0.85243 [0.28881]	0.43928 [0.0021146 **]
454	454_F341	-8.7961 [1.648e-24 ***]	1.7896 [0.10775]	0.64841 [0.0010465 **]
454	454_816R1	-8.9712 [1.9378e-15 ***]	-0.49231 [0.73315]	0.69347 [0.0082033 **]
PB	PBv1F	-6.8797 [2.068e-17 ***]	0.33488 [0.078993 .]	0.34099 [0.035795 *]
PB	PBv9R	-6.8797 [2.068e-17 ***]	0.33488 [0.078993 .]	0.34099 [0.035795 *]
